# Band asymmetry–driven nonreciprocal electronic transport in a helimagnetic semimetal α-EuP_3_

**DOI:** 10.1073/pnas.2405839122

**Published:** 2025-01-23

**Authors:** Alex Hiro Mayo, Darius-Alexandru Deaconu, Hidetoshi Masuda, Yoichi Nii, Hidefumi Takahashi, Rodion Vladimirovich Belosludov, Shintaro Ishiwata, Mohammad Saeed Bahramy, Yoshinori Onose

**Affiliations:** ^a^Institute for Materials Research, Tohoku University, Sendai 980-8577, Japan; ^b^Department of Physics and Astronomy, School of Natural Sciences, The University of Manchester, Manchester M13 9PL, United Kingdom; ^c^Division of Materials Physics, Graduate School of Engineering Science, Osaka University, Toyonaka 560-8531, Osaka, Japan

**Keywords:** nonreciprocal electronic transport, chirality, helimagnetic semimetal

## Abstract

Chiral magnetic textures give rise to unconventional magnetotransport phenomena such as the topological Hall effect and nonreciprocal electronic transport. While the correspondence between topology or symmetry of chiral magnetic structures and such transport phenomena has been well established, a microscopic understanding based on the spin-dependent band structure in momentum space remains elusive. Here, we demonstrate how a chiral magnetic superstructure introduces an asymmetry in the electronic band structure and triggers a nonreciprocal electronic transport in a centrosymmetric helimagnet α-EuP_3_. The magnetic structure of α-EuP_3_ is highly tunable by a magnetic field and closely coupled to its semimetallic electronic band structure, enabling a systematic study across chiral and achiral magnetic phases on the correspondence between nonreciprocal transport and electronic band asymmetry. Our findings reveal how a microscopic change in the magnetic configuration of charge carriers can lead to nonreciprocal electronic transport, paving the way for designing chiral magnets with desirable properties.

## Significance

Chirality in materials can encode information using its two distinguishable enantiomers as binary states. In helimagnets, nonreciprocal electronic transport offers a potential readout method by mirroring the material’s chirality. Yet, the underlying mechanisms remain poorly understood, limiting the application of helimagnetic chiral information. The current understanding primarily relies on phenomenological observations of real-space dynamics, rather than on a microscopic examination in momentum space. Our study reveals that the chiral magnetic texture induces an asymmetry in the electronic band structure, triggering nonreciprocity. This finding provides crucial insight into the interplay between real space and momentum space, indispensable for designing spintronic functionalities in chiral magnets.

Chirality is the breaking of mirror symmetry. The concept of chirality is applicable in a wide range of scientific fields, from high-energy physics to biology (1). In condensed matter, the chirality caused by the crystal structure is primarily studied. However, there is a distinct class of materials where the magnetic structure induces chirality. In particular, in helimagnets, the spiral arrangement of magnetic moments breaks the mirror symmetry, thus inducing chirality. The topological magnetic textures denoted as a skyrmion lattice can be viewed as the superposition of several helimagnetic structures (2, 3). The chiral magnetic textures give rise to a number of unique physical properties, among which the nonreciprocal electronic transport has been extensively studied recently (4–7).

Nonreciprocal electronic transport is the rectification of electric conduction in systems without spatial-inversion (P) and time-reversal (T) symmetries (8–12). When the crystal structure breaks P, the mechanism is well understood. For example, in BiTeBr (13), the polar crystal structure induces a Rashba-type spin splitting in the electronic structure. Then, an applied magnetic field gives rise to the asymmetry of the electronic band structure, causing a rectification effect. On the other hand, in chiral magnetic textures with a centrosymmetric crystal structure, it is the arrangement of the spins that breaks P in a distinct manner such that it gives rise to a finite chirality in the system. The nonreciprocal electronic transport has been studied in chiral magnets such as MnP (6) and MnAu_2_ (7), showing a sizeable effect of magnetic chirality on electronic transport. However, the mechanism of nonreciprocal electronic transport in helimagnetic systems remains unclear.

**Fig. 1.**
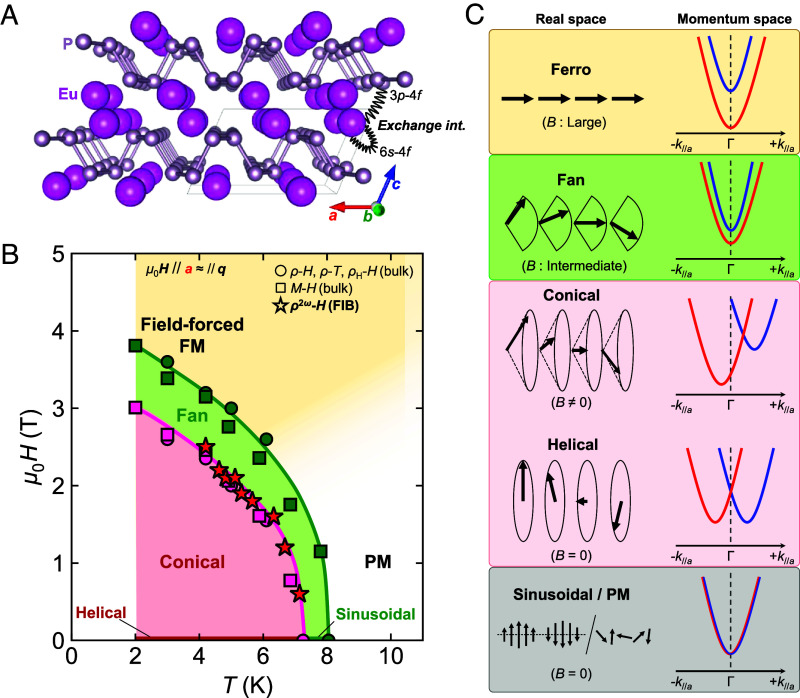
Crystal, magnetic, and expected band structures in α-EuP_3_. (*A*) Crystal structure of α-EuP_3_. The localized Eu-4*f* moments couple with the itinerant electrons in the system, which consist of P-3*p* and Eu-6*s* orbitals. (*B*) Field-temperature phase diagram of α-EuP_3_ for a magnetic field *μ*_0_***H***//***a***, approximately aligned with the propagation vector ***q*** of the magnetic phases. (*C*) Schematic illustrations of the symmetrically expected relationship between the magnetic structure and the momentum-space band structure. Although the band structures are symmetric under sinusoidal, paramagnetic, helical, fan, and ferromagnetic structures, the conical magnetic structure induced by a moderate magnetic field applied along the ***q***-vector gives rise to an asymmetrical deformation of the band structure along the *k*-path in the ***q***-vector direction.

In this article, through a combination of nonreciprocal electronic transport experiments and first-principles band structure calculations, we demonstrate that the nonreciprocal electronic transport is induced by a magnetically driven asymmetry of the band structure in a helimagnetic semimetal α-EuP_3_. α-EuP_3_ has a centrosymmetric crystal structure with a monoclinic space-group type *C*2/*m* (14), characterized by a single mirror plane perpendicular to the twofold rotational *b*-axis (Fig. 1*A*). The 4*f*-states of the divalent Eu ions are highly localized and relatively close in energy to the Fermi level and thus behave as local magnetic moments interacting through carrier-mediated Ruderman–Kittel–Kasuya–Yosida coupling (15–17).

## Results and Discussion

It has been recently noted that α-EuP_3_ exhibits a characteristic interplay between magnetism and electronic structure, leading to unusual magnetotransport responses such as a large anomalous Hall effect caused by a topological transition (14). However, the discussion was mainly for the paramagnetic regime, and therefore the transport involving the magnetically ordered regime remains unraveled. Fig. 1*B* shows the field-temperature phase diagram of α-EuP_3_ based on resistivity and magnetization measurements under magnetic fields applied along the *a*-axis. Two low-temperature magnetic phases are observed, consistent with the previously reported results of isostructural P-rich Eu(As_1-_*_x_*P*_x_*)_3_ (18–21). The system undergoes two incommensurate phases upon decreasing temperature: a sinusoidal phase and, at lower temperatures, a helimagnetic phase. Both phases have almost the same magnetic propagation vector [***q*** ~ (−0.726, 0, 0.255) and (−0.726, 0, 0.222), respectively (18–20)], nearly parallel to the crystalline *a*-axis. Under an applied magnetic field along the *a*-axis, approximately perpendicular to the helical plane, the magnetic structure first forms a conical structure. As the field increases, it is naturally expected that at some point, the cone collapses into a fan structure (22), where the moment along the *c*-axis vanishes and leaves an oscillating component along the magnetically easy *b*-axis. Eventually, this evolution ends in a field-forced ferromagnetic (FM) configuration.

The above process is schematically shown in Fig. 1*C*. As can be seen, the breaking of P in the helimagnetic state forbids any energy degeneracy between an electronic state *E*(*k_q_*, *s_q_*) and either *E*(-*k_q_*, *s_q_*) or *E*(*k_q_*, -*s_q_*), where *k_q_* and *s_q_* are the momentum and spin components, respectively, along the helimagnetic propagation vector ***q***. This results in a symmetric spin-polarized band structure. Applying a magnetic field along ***q***, where the conical magnetic structure forms, introduces an asymmetry in the band structure, which, as we will show later, leads to nonreciprocal behavior in electronic transport. When the magnetic field is strong enough, the band structure becomes symmetric again with a fan-shaped achiral magnetic structure. Eventually, all spins are fully aligned with the magnetic field, leading to an FM structure. The variation of the band structure in the course of magnetic phase transitions was partly discussed in a simplified theoretical model (23) but has never been observed in a real material.

The resistivity in helimagnets along the propagation vector can be described up to the nonlinear regime as

[1] $$\rho \left(j\right)\;=\;\rho_{1}\;+\;\rho_{2}j\;+\;\cdot \cdot \cdot ,$$

where *ρ*_1_ (=*ρ*^1^*^ω^*) and *ρ*_2_ reflect the ordinary resistivity and nonlinear resistivity, respectively, and *j* is the electric current density. The second term can be sensitively measured as a second-harmonic voltage under an ac electric current *j* = *j*_0_ sin*ωt* (8);

[2] $$E=\rho^{1\omega }j_{0}\sin\omega t+\rho^{2\omega }j_{0}(1\;–\;\cos2\omega t)+\cdot \cdot \cdot .$$

**Fig. 2.**
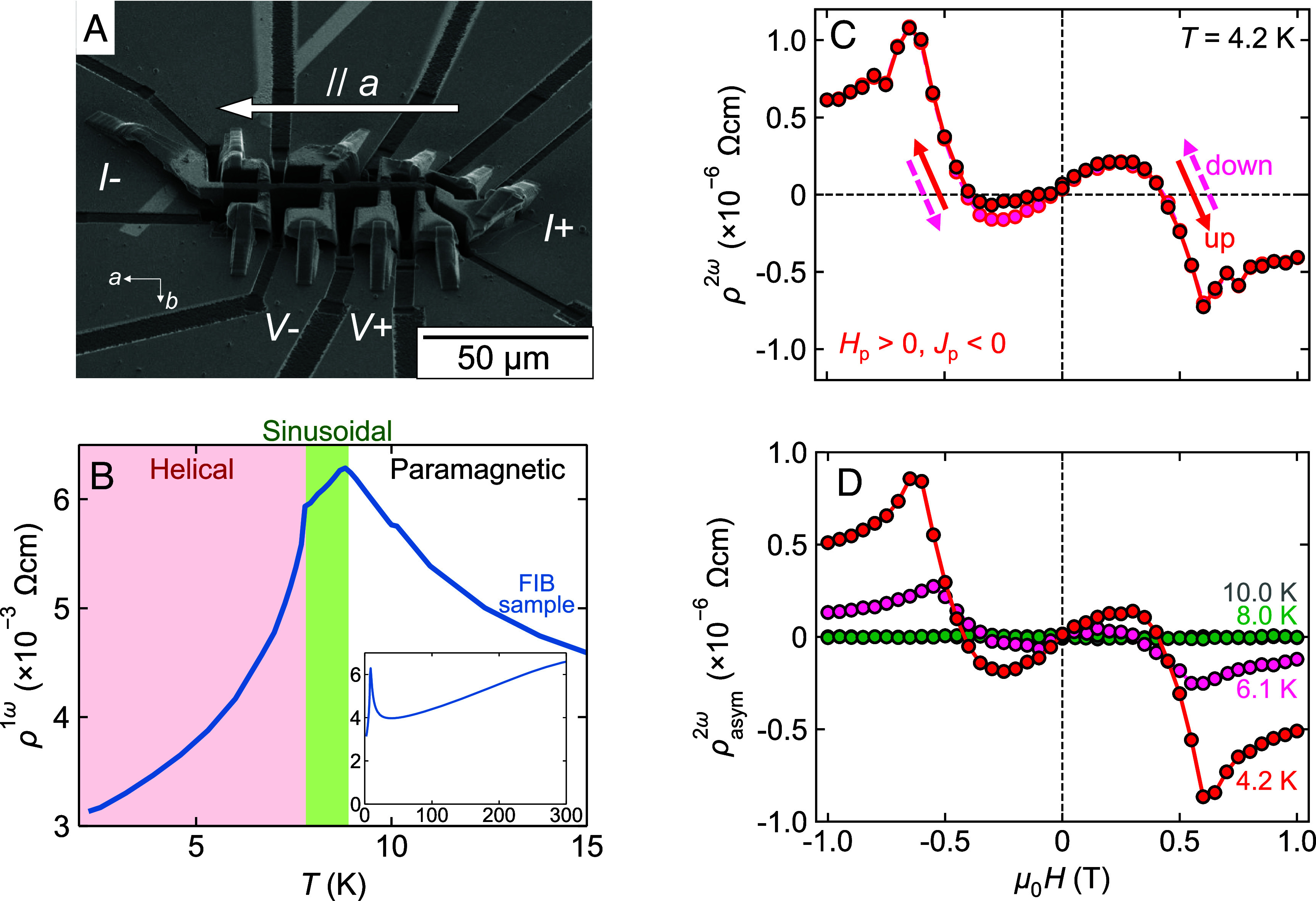
Nonreciprocal electronic transport measured in a microfabricated α-EuP_3_ sample. (*A*) Scanning electron microscope (SEM) image of the microfabricated α-EuP_3_ sample by the FIB technique. (*B*) Temperature dependence of resistivity near the magnetic transition temperatures for the microfabricated sample. The *Inset* shows the resistivity data up to room temperature. (*C*) Magnetic field dependence of the second-harmonic resistivity *ρ*^2^*^ω^* after the attempt of chirality control with antiparallel magnetic field and electric current (*H*_p_ > 0, *J*_p_ < 0). The red (pink) marker indicates the field increase (decrease) sweep. (*D*) Temperature dependence of the field-asymmetric component of the second-harmonic resistivity *ρ*^2^*^ω^*_asym_. The data shown represent the maximum response obtained among the *H*_p_ > 0, *J*_p_ < 0 chirality control attempts.

*ρ*^2^*^ω^* = *ρ*_2_*j*_0_/2 is composed of field-symmetric and field-asymmetric components as *ρ*^2^*^ω^ = ρ*^2^*^ω^*_sym_ + *ρ*^2^*^ω^*_asym_, where *ρ*^2^*^ω^*_sym_(+*H*) = *ρ*^2^*^ω^*_sym_(−*H*) and *ρ*^2^*^ω^*_asym_(+*H*) = −*ρ*^2^*^ω^*_asym_(−*H*). *ρ*^2^*^ω^*_asym_ is caused by the nonreciprocal electronic transport while *ρ*^2^*^ω^*_sym_ may be induced by some extrinsic effect such as the effect of electrode contact. Because $\rho^{2\omega }\propto j_{0}$, a large electric current density *j*_0_ is preferred for the measurement of nonlinear electronic transport. To realize a large current density, the α-EuP_3_ single crystal sample was microfabricated into a device with a cross-sectional area of ~8 × 4 μm^2^ (Fig. 2*A*), using the focused ion-beam (FIB) technique (24). The temperature dependence of resistivity for the FIB sample clearly shows two magnetic transitions at low temperatures (Fig. 2*B*). The overall resistivity behavior is consistent with previously reported bulk sample results (14), assuring the device quality.

Fig. 2*C* exemplifies the second-harmonic resistivity *ρ*^2^*^ω^* as a function of the magnetic field at 4.2 K, measured by a lock-in amplifier. *ρ*^2^*^ω^* shows an evident field-asymmetric behavior with minimal hysteresis. *ρ*^2^*^ω^* seems mostly composed of an intrinsic nonreciprocal electronic resistivity *ρ*^2^*^ω^*_asym_, which is induced by the finite chirality of the system arising from helimagnetic order. Fig. 2*D* shows the field asymmetric component *ρ*^2^*^ω^*_asym_ = [*ρ*^2^*^ω^_H_*_-increase_(+*H*) − *ρ*^2^*^ω^_H_*_-decrease_(−*H*)]/2, where the subscripts of *ρ*^2^*^ω^_H_*_-increase_ and *ρ*^2^*^ω^_H_*_-decrease_ describe the sweeping direction of the magnetic field. Since *ρ*^2^*^ω^* was measured for a full loop of 0 T → +1 T → −1 T → 0 T, *ρ*^2^*^ω^*_asym_ is presented in quadrants of both positive and negative magnetic fields. Note that the sign and magnitude of *ρ*^2^*^ω^* are not reproduced in repeated measurements, presented in detail in Supporting Information (*SI Appendix*, Fig. S2). This is because there are two energetically degenerate chiral states in the centrosymmetric crystal α-EuP_3_, and the chiral domain population differs in each measurement. Recent studies on centrosymmetric helimagnetic metals with Mn magnetic moments have reported that the helimagnetic chirality can be controlled by traversing the achiral–chiral transition while simultaneously applying a magnetic field and a high-density dc electric current (6, 7, 25, 26). The sign of the controlled chirality depends on whether the magnetic field and dc current are parallel or antiparallel. Following these reports, we have attempted to control the chirality by a similar procedure with the magnetic field *H*_p_ and dc current *J*_p_ before the measurement of *ρ*^2^*^ω^* shown in Fig. 2*C* and *SI Appendix*, Fig. S2 (for the details of the procedure, see *SI Appendix*). As shown in *SI Appendix*, Fig. S2*A*, the sign of *ρ*^2^*^ω^*_asym_ does not depend on *H*_p_ and *J*_p_ but seems random, indicating the control procedure was not effective in α-EuP_3_ (for the reasons of ineffectiveness, see *SI Appendix*, Section 1). Nevertheless, the magnetic field evolution was generally reproducible. Notably, *ρ*^2^*^ω^*_asym_ shows a complex magnetic field dependence. As the magnetic field is increased from 0 T, it first shows a broad peak and then steeply changes accompanying a sign change followed by a sharp peak. Fig. 2*D* shows the magnetic field dependence of *ρ*^2^*^ω^*_asym_ measured at temperatures of 4.2 K, 6.1 K, 8.0 K, and 10.0 K. The *ρ*^2^*^ω^* measurements were done several times at each temperature (*SI Appendix*, Fig. S2), and the presented data in Fig. 2*D* correspond to the maximum response obtained. *ρ*^2^*^ω^*_asym_ distinctly demonstrates nonreciprocity only within the chiral magnetic phase (4.2 K and 6.1 K) and vanishes in the achiral phases (8.0 K and 10.0 K). These results cannot be explained by the extrinsic heating mechanism as detailed in SI Appendix and clearly show the correspondence between *ρ*^2^*^ω^*_asym_ and the magnetic symmetry.

**Fig. 3.**
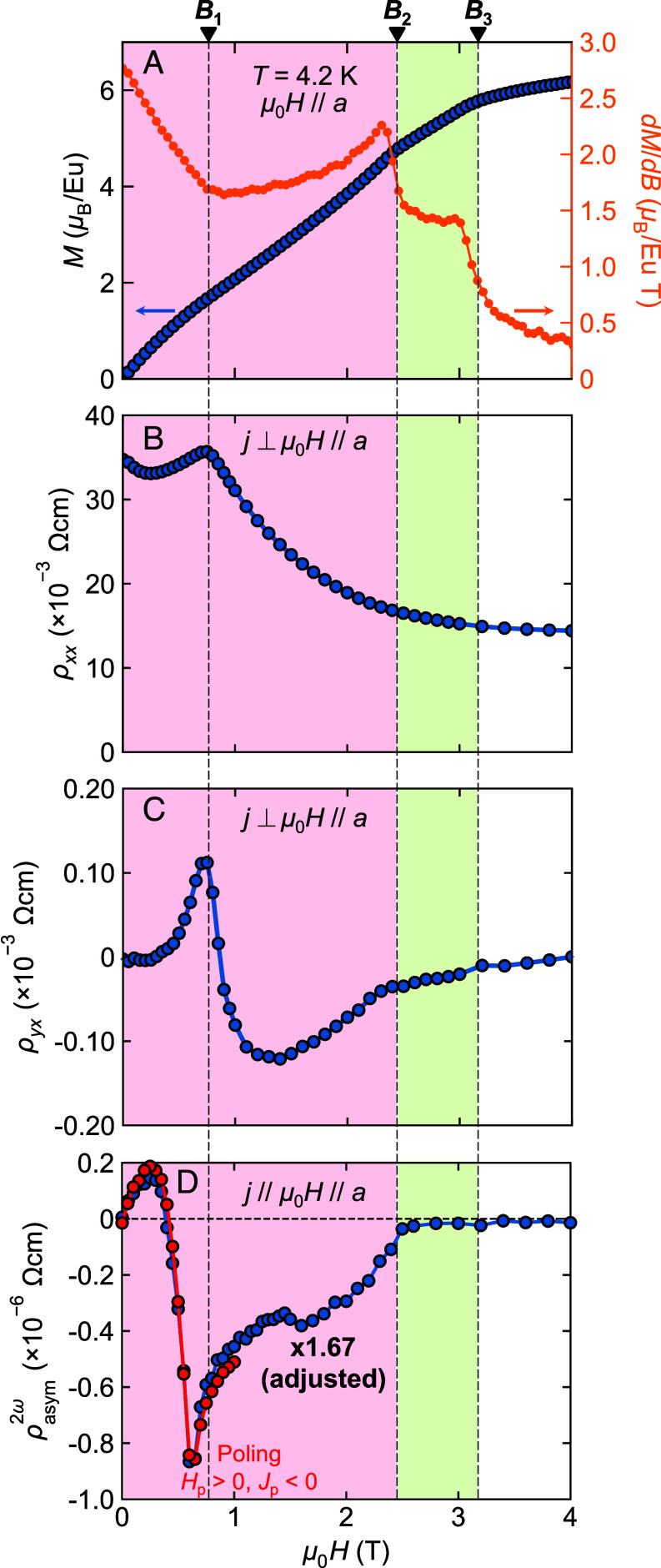
Magnetic field dependence of transport and magnetic properties across chiral-achiral phase transitions. Magnetic field dependence of the (*A*) magnetization *M* and its field derivative, (*B*) longitudinal resistivity *ρ_xx_*, (*C*) transverse (Hall) resistivity *ρ_yx_*, and (*D*) *ρ*^2^*^ω^*_asym_, at *T* = 4.2 K under *μ*_0_***H***//***a*** up to 4 T. (*A*–*C*) were measured in bulk samples and (*D*) was measured in the FIB sample. *ρ*^2^*^ω^*_asym_ shown in blue in panel *D* was measured without attempting chirality control. The magnitude is adjusted to match the maximum value presented in Fig. 2*D*.

Let us now examine the detailed magnetic field dependence of the *ρ*^2^*^ω^*_asym_ signal across chiral–achiral transitions. Fig. 3*D* displays the nonreciprocal electronic response at 4.2 K while sweeping the magnetic field from +4 T to −4 T without any chirality-control procedure, with the data antisymmetrized as *ρ*^2^*^ω^*_asym_ = [*ρ*^2^*^ω^*(+*H*) − *ρ*^2^*^ω^*(−*H*)]/2. This antisymmetrization is valid when the magnetic hysteresis is negligible, as confirmed in Fig. 2*C*. The obtained data are scaled to that shown in Fig. 2*D* by multiplying a constant. The *ρ*^2^*^ω^*_asym_ signal shows a sharp peak around *B*_1_ ~ 0.7 T. In the high field region, *ρ*^2^*^ω^*_asym_ gradually decreases and vanishes at around *B*_2_ ~ 2.5 T. For comparison, we plot the magnetic field dependences of magnetization *M* and its field derivative, longitudinal resistivity *ρ_xx_*, and Hall resistivity *ρ_yx_* in Fig. 3 *A*–*C*. Clear anomalies at *B*_2_ and *B*_3_ ~ 3.1 T correspond to the transition from conical to fan and from fan to field-forced FM phases, respectively. The disappearance of *ρ*^2^*^ω^*_asym_ at *B*_2_ is again in excellent agreement with the magnetic symmetry (marked as red stars in Fig. 1*B*). In addition, a slight anomaly is discerned in the derivative of magnetization around *B*_1_. In contrast, this *B*_1_ anomaly is conspicuously observed in the longitudinal and Hall resistivities, both showing a peak. In a previous paper, Mayo et al. (14) reported a Fermi surface reconstruction induced by the FM polarization of Eu 4*f* moments, occurring at an approximate threshold magnetization of *M* ~ 1.8 μ_B_/Eu. The observed anomalies at *B*_1_ indeed correspond to a similar magnetization as the reported threshold. The fact that the anomaly is notable in the transport properties and less visible in magnetization suggests a strong role of the Fermi surface in the observed nonreciprocity (for further notes on magnetotransport results, see *SI Appendix*).

**Fig. 4.**
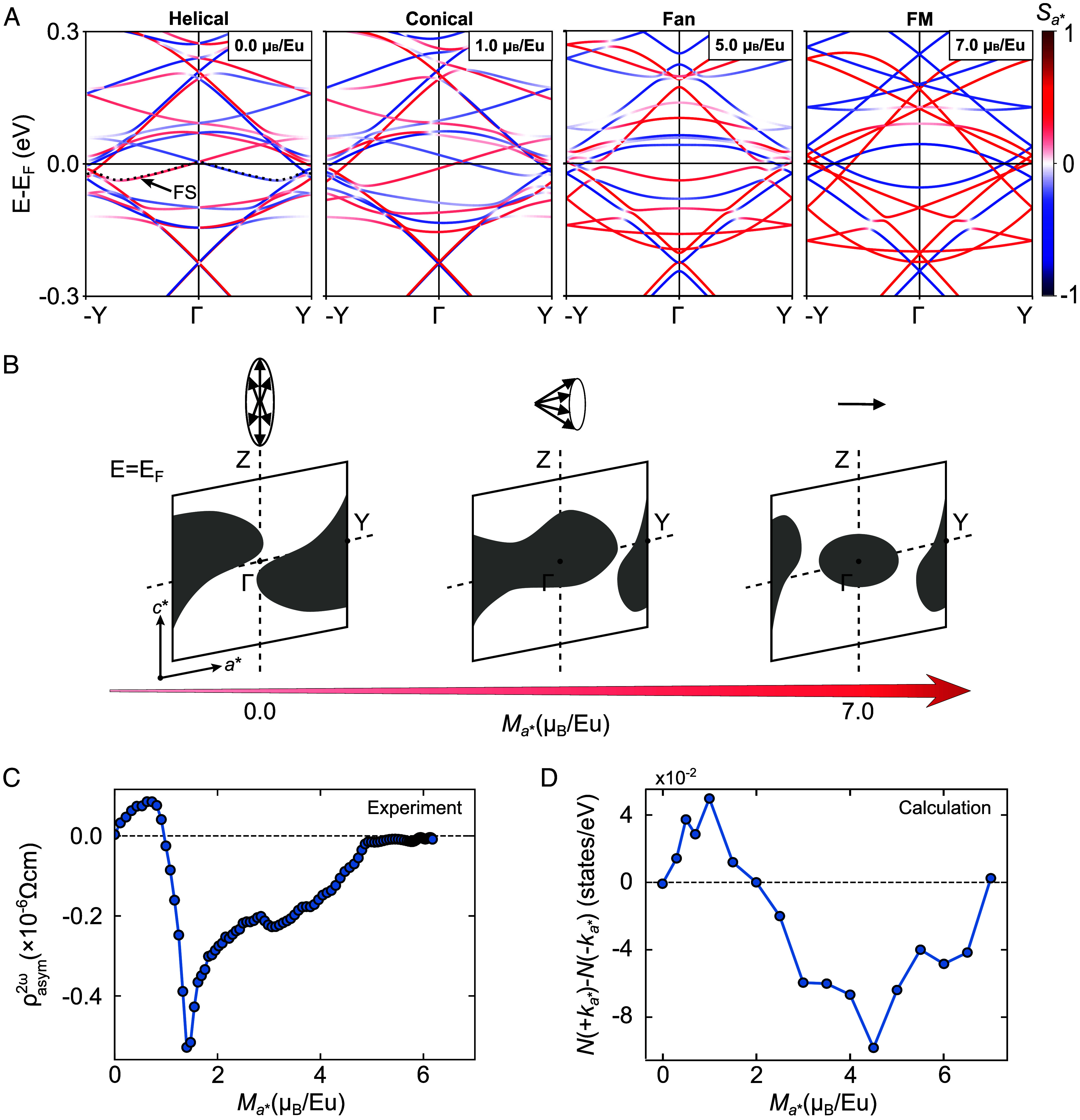
Electronic band structures of α-EuP_3_ in each magnetically ordered state. (*A*) The electronic band structures in the helimagnetic, conical, fan, and field-forced ferromagnetic (FM) states, respectively. The calculation was carried out by tilting the Eu magnetic moments along the *a*-axis and imposing the magnetic structures corresponding to the experimental setup of *μ*_0_***H***//***a***. (*B*) Schematic of the field evolution of the Fermi pockets derived from a single-electron band denoted in panel *A* in the conical magnetic state. (*C*) Magnetization dependence of experimental *ρ*^2^*^ω^*_asym_. (*D*) Magnetization dependence of the difference in the calculated density of states between +k_a__*_ and −k_a__*_ in the conical magnetic state.

To understand how the experimentally observed nonreciprocity in α-EuP_3_ arises from microscopic changes in its band structure, we have conducted first-principles band calculations for the relevant magnetic structures: helical, conical, fan, and FM (*Materials and Methods* and *SI Appendix*). We construct each magnetic phase by appropriately constraining the directions of Eu magnetic moments in a sufficiently large supercell as shown in *SI Appendix*, Fig. S8. It is to be noted that the reported helical magnetic order is incommensurate and slightly tilted from the crystalline *a*-axis [***q*** ~ (−0.726, 0, 0.222)]. We have instead considered a commensurate helimagnetic model with ***q*** = (−0.75, 0, 0) and a helical plane perpendicular to ***q*** for the sake of simplicity. The validity of this simplification is examined in *SI Appendix*, section 7. To model the intermediate phases induced by the magnetic field, the Eu magnetic moments were gradually tilted along the *a*-axis through a systematic linear interpolation between the helimagnetic and ferromagnetic phases. This resulted in a net magnetization along the *a*-axis with a conical magnetic configuration, which we used to find its corresponding magnetic field in our experimental setup *μ*_0_***H***//***a*** in this phase. The fan structure was separately constructed by setting the c*-axis (c*//***a*** × ***b***) components of the magnetic moments to zero and collapsing the conical structure. Fig. 4*A* displays the band structure calculated for each of these magnetic phases along the −Y – Γ – +Y direction, corresponding to the a*-axis direction (a*//***b*** × ***c***). As can be seen, in the helical phase (*M* = 0 μ_B_/Eu), the spin degeneracy is lifted due to a spin-dependent shift along the ±Y direction rather than a Zeeman-like splitting, characteristic to a *ρ*-broken system. It should be noted that the momentum-dependent spin splitting seen here differs from the Rashba-type, in which the shifted direction in momentum space is perpendicular to the spin direction. Rather, the spin direction of the split bands is parallel to the direction of momentum shift, similar to the case of chiral materials such as Te (27). Nonetheless, the overall band structure is symmetric along −Y – Γ – +Y.

Upon introducing a conical deformation in the magnetic structure, achieved through applying a relatively weak magnetization along the *a*-axis, a notable transformation unfolds within the band structure, evident as a pronounced asymmetry in the band dispersions along −Y – Γ – +Y. This asymmetry implies a discernible distinction in the charge current induced by an electric field +*E* as compared to that of *−E*. Consequently, the system is anticipated to manifest a finite nonreciprocal electronic resistivity within the conical phase. Remarkably, as the magnetic phase transitions away from the conical phase into the achiral fan magnetic or ferromagnetic phase, a restoration of symmetry becomes apparent in the band structure along −Y – Γ – +Y, as illustrated in Fig. 1*C*. This intriguing finding provides compelling evidence as to why nonreciprocal electronic transport is exclusively observable in the conical magnetic structure and explains how this distinct asymmetry in the band dispersions is responsible for it.

To further discuss the effect of field variation in the conical magnetic state, we turn our attention to the conical angle dependence of the band structure. Fig. 4*B* schematically illustrates the conical angle-dependent evolution of the relevant Fermi pockets. Here, we show the pockets corresponding to the energy level indicated by the dashed lines in Fig. 4*A*. As can be seen, they appear as two symmetric pockets in the helimagnetic state, as expected. However, upon tilting the magnetic moments along the *a*-axis and, hence, increasing the conical angle, an asymmetric deformation of the energy pockets emerges. In the FM state, the energy pockets regain symmetry.

Another notable feature seen in the experiment is a sharp sign change followed by a negative peak in *ρ*^2ω^_asym_ under increasing magnetization (Fig. 4*C*). As a measure of band asymmetry, we plot the residual density of states, defined as the difference between the total density of states for +k_a__*_ and that for −k_a__*_, in the conical phase as a function of conical magnetization in Fig. 4*D*. As can be seen, it initially shows a positive value followed by a sign change and a discernible negative peak. This behavior is consistent with the experimental observation of the nonreciprocal resistivity (Fig. 4*C*). Despite its apparent simplicity, it is remarkable that our theoretical model not only captures but also qualitatively agrees with the experiment, further providing compelling evidence that the nonreciprocal electronic transport is induced by the asymmetry in the momentum-space electronic bands.

In summary, we have investigated nonreciprocal electronic transport in a magnetic semimetal α-EuP_3_. The material facilitates successive tunability of the magnetic structure from helimagnetic to induced ferromagnetic structures via conical and fan structures in response to a magnetic field, presenting a rich array of transport responses coupled with the magnetic phases. The nonreciprocal electronic transport was found to arise as the chiral magnetic structure imposes an asymmetric deformation in the electronic band structure and vanishes as the magnetic structure is tuned into an achiral phase, restoring symmetry in momentum space. Moreover, a singular sign change of nonreciprocal electronic transport in the middle of the conical phase is observed, seemingly caused by the reversal of band asymmetry in the course of the dramatic exchange splitting inherent in this material. These results unravel that the nonreciprocal electronic transport in helimagnets originates from the band asymmetry caused by the magnetic chirality. The modeling of nonlinear transport on the Boltzmann level for this material remains an open challenge at present.

The relation between the band asymmetry and the nonreciprocal electronic transport was first suggested for a semiconductor with a noncentrosymmetric crystal structure (13). The present work shows that this relation is valid also for helimagnets. Since long-period chiral magnetic structures can be viewed as the superposition of helimagnetic and sinusoidal structures (28), the present result can be generalized to other chiral spin structures such as the skyrmion lattice. Moreover, similar relations have recently been studied in symmetry-broken correlated states. The asymmetry originating from the momentum-dependent orbital moment is proposed to induce the nonreciprocal transport in the charge-ordered Kagome metal CsV_3_Sb_5_ (29). One of the major mechanisms of the superconducting diode effect is finite-momentum Cooper pairing, induced by band asymmetry originating from the lack of time-reversal and inversion symmetries (30–35). The relation between momentum-space asymmetry and nonreciprocal transport appears to be quite generic.

## Materials and Methods

### Single Crystal Growth and Crystal Identification.

Single crystals of α-EuP_3_ were grown by the high-pressure synthesis method (14). The crystal was identified as α-EuP_3_ by powder and single-crystal X-ray diffraction techniques.

### FIB-Microfabrication of Transport Devices.

The Hall bar device was fabricated using the FIB technique (24) in the following steps: first, a small piece of single-crystal α-EuP_3_ with an approximate size of 100 × 50 × 8 μm^3^ was placed on an oxidized single-crystal silicon wafer. Next, 20 nm of Pt was deposited by RF magnetron sputtering, followed by 200 nm of Au deposited by electron beam evaporation, both covering the entire sample. The sample was then fixed to the substrate by FIB-assisted Pt deposition, ensuring electrical contact between the sample and substrate electrodes, and then was patterned into a Hall bar by FIB. Finally, the Pt/Au layer on the central top surface of the Hall bar channel was etched away by FIB. Additional information can be found in *SI Appendix*, section 12.

### Magnetotransport Measurements.

For the bulk measurements, electrode contacts were made by depositing 200 nm of Au on the sample and connecting Au wires using silver paste (*SI Appendix*, Fig. S4). Both bulk and FIB samples were subjected to transport measurements in a superconducting magnet. The ac resistivities were measured using the four-wire configuration and the standard lock-in technique, applying an ac electric current with a frequency of 11.15 Hz. The ac electric current density applied for the second-harmonic resistivity measurement in the FIB sample was set to an amplitude of 1 × 10^7^ A/m^2^.

### Band Structure Calculations.

The electronic structure calculations were performed within density functional theory (DFT) using the Perdew–Burke–Ernzerhof (PBE) exchange-correlation functional (36) as implemented in the VASP program (37, 38). To properly treat the strong on-site Coulomb interaction of Eu-4*f* states, an effective Hubbard-like potential term *U*_eff_ was added to the PBE functional. The value of *U*_eff_ was fixed at 6 eV for Eu-4*f* orbitals (39) and zero for all other orbitals. Relativistic effects including spin–orbit coupling were fully taken into account. The cutoff energy for plane waves forming the basis set was set to 400 eV. The lattice parameters and atomic positions were taken from experiment (14). The corresponding Brillouin zone for the 4 × 1 × 1 supercell (main text) and 4 × 1 × 2 supercell (*SI Appendix*) calculations were sampled using a 2 × 8 × 8 and 2 × 8 × 4 *k*-mesh, respectively. The calculation for each magnetic structure was carried out by imposing the corresponding magnetic moment configurations on the Eu site. The density-of-states calculations were performed using a 64-band effective Hamiltonian, downfolded from the DFT calculations using maximally localized Wannier functions (40). The sampling of the corresponding Brillouin zone was done using a 240 × 240 × 240 *k*-mesh.

## Supplementary Material

Appendix 01 (PDF)

## Data Availability

All study data are included in the article and/or *SI Appendix*.
