# Supplementary material for: Band asymmetry–driven nonreciprocal electronic transport in a helimagnetic semimetal α-EuP3
Source: Proc Natl Acad Sci U S A. 2025 Jan 23;122(4):e2405839122. doi: 10.1073/pnas.2405839122 (PMC11789083; doi:10.1073/pnas.2405839122)
Supplement: Supplementary file 1 — Appendix 01 (PDF) [file pnas.2405839122.sapp.pdf]

Supporting Information for
Band-asymmetry-driven nonreciprocal electronic transport in a helimagnetic semimetal α-EuP_3

Alex Hiro Mayo[a]*, Darius-Alexandru Deaconu[b], Hidetoshi Masuda[a], Yoichi Nii[a], Hidefumi Takahashi[c], Rodion Vladimirovich Belosludov[a], Shintaro Ishiwata[c], Mohammad Saeed Bahramy[b], and Yoshinori Onose[a]

*To whom correspondence may be addressed: Alex Hiro Mayo
Email: alex.hiro.mayo.d1@tohoku.ac.jp

This PDF file includes:

Supporting text
Figures S1 to S21
Tables S1
SI References

Supporting Information Text

Section 1. Multiple trials of the chirality-control procedure

Figure S1 schematically illustrates the relation between the chirality-control procedure and the magnetic phases in α-EuP_3.

In a centrosymmetric helimagnet, the opposing chirality are energetically degenerate and therefore the system will basically form magnetic domains of opposite chirality when undergoing helimagnetic ordering. In a recent study, Jiang *et al.*[6] reported that the helimagnetic chirality in a centrosymmetric crystal can be controlled by traversing the achiral-chiral transition while simultaneously applying a magnetic field and a high-density dc electric current, which they referred to as the "poling" procedure. The simultaneous application of the magnetic field and the electric current lifts the degeneracy relevant to the chirality and energetically favors one chirality over the other, depending on whether the magnetic field and dc current are parallel or antiparallel. A sufficiently large electric current density is essential for this procedure; thus, microfabrication of the sample is again vitally important. Jiang *et al.* achieved chirality-control in centrosymmetric MnP, controlling the system into a single chirality domain. A constant dc electric current density in the order of 1×10^9 A/m^2 was applied while sweeping the magnetic field, traversing the achiral-chiral phase boundary. It was also confirmed that the chirality can be switched to the opposite sign depending on the parallel/antiparallel application of the magnetic field and electrical current. The effectiveness of the poling procedure was also demonstrated in another Mn-based centrosymmetric metal $MnAu_2$[7].

Following these reports, we have attempted to control the chirality by a similar procedure with the magnetic field H_p and dc current J_p before the measurement of $\rho^{2\omega}$. Because the current induced heating is significant at the low temperature region, we utilized the sample's Joule heating to traverse the magnetic phases, as an alternative to sweeping the chamber temperature. Figure S1 illustrates how the system traverses through magnetic phases during the poling procedure in this work. A magnetic field of H_p = 1.5 T was first applied. Then, the magnitude of the dc electric current density was slowly applied up to $J_p = \pm1\times10^8$ A/m^2, traversing the magnetic phases and resulting in the paramagnetic (PM) phase due to Joule heating. In contrast to previous works [6,7], where the magnetic field was swept, the magnetic field was fixed in the present work, and the dc electric current was swept. During the process, the sample temperature was monitored via the sample resistance, and we confirmed that the temperature was well above the higher transition temperature of 8K when the dc electric current reached $\pm1\times10^8$ A/m^2. After slowly sweeping the dc current back down to zero, undergoing successive magnetic transitions from PM to fan and from fan to conical, the magnetic field was quenched. Then, $\rho^{2\omega}$ was measured by sweeping the magnetic field in a loop from 0 T → +1 T → -1 T → 0 T, ensuring the system stays well within the conical phase and that the state prepared by the poling procedure was preserved. This procedure with $H_p > 0$, $J_p < 0$

was executed prior to the measurement presented in Fig. 2C. Same measurements were repeated three times each with $H_p > 0$, $J_p < 0$ (antiparallel poling, shown in red) and with $H_p > 0$, $J_p > 0$ (parallel poling, shown in blue), presented in Figure S2. The grey arrows in Figure S1 describe the path the system undergoes during the poling procedure in this work.

Figure S2 shows the multiple trials of the poling procedure followed by a second-harmonic resistivity measurement. Same measurements were repeated three times each with $H_p > 0$, $J_p < 0$ (antiparallel poling, shown in red) and with $H_p > 0$, $J_p > 0$ (parallel poling, shown in blue) for each temperature (T = 4.2 K, 6.1 K, 8.0 K, 10.0 K). In Figure 2D in the main text, the data showing a large response among the $H_p > 0$, $J_p < 0$ poling attempts in each of the following panels is selected and displayed.

In stark contrast to the manganese helimagnets[6, 7], the control with magnetic fields and electric current appears to be ineffective in α-EuP_3. One possible reason for this is the small magnitude of J_p. $|J_p| = 1\times10^8$ A/m^2 applied here is close to the minimum value for successful chirality-control in the case of manganese helimagnets[6, 7]. Another reason can be related to the orbital character of the Fermi surface. In the manganese metals, Mn 3*d* orbitals have a large density of states contributing to the Fermi surface[S1], whereas in α-EuP_3, the P-3*p* orbitals at the Fermi surface and the Eu-4*f* states are only weakly hybridized[14], possibly resulting in a weaker momentum-transfer between the electric current and the local moments during the poling procedure[22, S2, S3, S4]. Although in theory one can expect that a larger $|J_p|$ should overcome the critical value and enable chirality control, the sample temperature readily exceeds the Néel temperature from Joule heating, limiting the parameter window to achieve controllability in α-EuP_3.

Section 2. Temperature dependence of $\rho^{2\omega}_{asym}$ – H and reproducibility of the nonreciprocal response in a different FIB device

Figure S3 displays $\rho^{2\omega}_{asym}$ measured in a different FIB sample from the one discussed in the main text. The device is shown in panel **A** and will be referred to as FIB sample No. 2 from here on. The electrode contacts were made by spot welding Au wires on a crystal. The crystal was then patterned into a Hall bar with a cross-sectional azrea of ~ 40 × 6 μm^2 by using the FIB technique. The temperature and magnetic field dependencies are quite similar to those presented in the main text, which confirms the reproducibility. The results from both FIB devices were used to plot the transition fields and the characteristic fields in Figure 1B and Figure S7, respectively.

Section 3. Description of the bulk measurements

Figure S4 shows the crystal orientation and measurement configuration for the bulk transport measurements presented in the main text. The crystal orientation is displayed in panels **A** and **B**. Electrode contacts were made by depositing 200 nm of Au on the sample. The sample was fixed on an oxidized single-crystal silicon wafer using varnish and Au wires were connected to the Au

electrodes using silver paste, as shown in panel **C**. The magnetic field was applied perpendicular to the sample, parallel to the crystalline *a*-axis.

Figures S5 and S6 show isotherms of the magnetic field dependence of magnetization and Hall resistivity, respectively. The low-field anomaly, denoted as B_1 in Fig. 3, is more evidently observed in the Hall effect rather than magnetization, indicating a Fermi-surface-related origin.

Section 4. Relationship between resistivity anomalies and magnetic phases

Figure S7 shows the magnetic phase diagram under $\mu_0\boldsymbol{H}$ // $\boldsymbol{a}$ in an extended temperature region up to 20 K. In addition to the information presented in Figure 1B, the following diagram displays the low-field anomalies (shown in black) observed in Figure 3. B_1, B_2, and B_3 which were denoted in Fig. 3 are denoted here as well. As noted in the main text, one can see in Fig. S7 that the critical points B_1 and B_2 observed in $\rho^{2\omega}{}_{\mathrm{asym}}$ agree well with the peaks or kinks also seen in the longitudinal and Hall resistivities and magnetization.

Although $\rho^{2\omega}{}_{\mathrm{asym}}$ is limited only within the chiral phase, a characteristic feature about the B_1-anomaly in the Hall resistivity extends into the PM region. The peak structure seen in Fig. 3C, although pronounced in the magnetically ordered phase, persists even in the PM region (Fig. S6). The B_1-anomaly throughout the whole temperature range is shown as a grey line in Fig. S7. Notably, the anomalies shown along the grey line correspond to the magnetization of 1.7 – 1.8 μ_B/Eu, which is the critical value where the Fermi surface reconstruction has been reported previously[14].

Section 5. First-principles calculations for the magnetic phases

Figure S8 shows the simplified models used in the electronic structure and density of states calculations presented in Figure 4.

The helimagnetic structure (Fig. S8A) was constructed based on the magnetic structure determined for isostructural P-rich $Eu(As_{1-x}P_x)_3$ (x = 0.80)[18]. The structure was slightly simplified for computability by making it a commensurate structure and aligning the propagation vector parallel to the *a*-axis, with components of $\boldsymbol{q}$ = (-0.75, 0, 0).

The conical structure (Fig. S8B) was then constructed by adding a component parallel to the *a*-axis and tilting the magnetic moments, corresponding to the experimentally applied magnetic field direction. The ferromagnetic structure (Fig. S8D) is an extrapolation of the conical model and corresponds to the high-field limit where finally all the magnetic moments align parallel to the *a*-axis.

The fan structure (Fig. S8C) was constructed by setting the *c**-axis component ($\boldsymbol{c}^*$ // $\boldsymbol{a} \times \boldsymbol{b}$, i.e., along the out-of-layer direction with respect to the layered crystal structure) to zero, resulting in a net magnetic moment along the *a*-axis and an oscillating component in the *b*-axis.

Section 6. Magnitude of the nonreciprocal electronic transport in reported polar and chiral systems

The nonlinear resistivity in $\mathscr{P}$-broken systems can be further described in detail as

$$\rho(\boldsymbol{B}, \boldsymbol{j}) = \rho_0 \, (1 + \gamma \boldsymbol{B} \cdot \boldsymbol{j} + \cdots)$$

in chiral systems, and

$$\rho(\boldsymbol{B}, \boldsymbol{j}) = \rho_0 \, [1 + \gamma (\boldsymbol{P} \times \boldsymbol{B}) \cdot \boldsymbol{j} + \cdots],$$

in polar systems, where ρ_0 is the linear (ordinary) resistivity at zero magnetic field and the second term represents the directional nonlinear resistivity[8]. ***B*** is the magnetic flux density and ***P*** is a unit vector parallel to the polarization vector. This nonlinear nonreciprocal transport in systems with broken $\mathscr{P}$ is named magnetochiral anisotropy (MCA)[9], and its magnitude can be characterized by the value of the MCA coefficient γ. In centrosymmetric systems, $\gamma = 0$, i.e., the MCA vanishes. Table S1 shows the comparison of the MCA magnitude among various $\mathscr{P}$-broken systems. Although the $|\gamma|$ value tends to be smaller in magnetic systems compared to materials where the crystal structure breaks $\mathscr{P}$, centrosymmetric α-EuP_3 exhibits MCA orders of magnitudes larger than other magnetic metals, even larger than some of the materials with $\mathscr{P}$-broken crystal structures. The exquisite coupling between the magnetic texture and the semimetallic small Fermi surface may be key in this observation, since a smaller Fermi energy will be closer in energy scale to the magnetic coupling, favorable for the large band deformation by the magnetic moments and also its detection via transport responses.

Section 7 Examination of the simplified magnetic structure in the theoretical model

In the band calculation, we adopt a simplified magnetic structure with the magnetic wave vector $\boldsymbol{q}$ = (-0.75, 0, 0) and the helical plane perpendicular to $\boldsymbol{q}$. The magnetic field and the electric current are assumed to be parallel to $\boldsymbol{q}$. In the following, we examine the validity of this simplification in the light of symmetry, $\boldsymbol{q}$-vector direction, and magnetic field direction.

(7-a) Symmetry

Here, let us compare helical and fan magnetic structures in the light of inversion symmetry. Fig. S9 depicts the effects of inversion symmetry operation on helical and fan magnetic structures. For the helimagnetic structure, spatial inversion converts the system to the opposite enantiomer (i.e., chirality reversal), showing broken $\mathscr{P}$ (Figs. S9A and B), therefore gives rise to the nonreciprocal electronic transport.

In contrast, we can see that for the fan structure (Fig. S9C), spatial inversion merely results in a translation of the original structure along the depicted *k*-direction (Fig. S9D) (In this example the translation is half a period). Therefore, in the light of magnetic point group, the fan structure is $\mathscr{P}$-invariant, which forbids nonreciprocal transport.

An important point is that this argument is valid for any $\boldsymbol{q}$-vector and helical plane. Therefore, the simplification does not matter for the symmetrical rules of nonreciprocal electronic transport.

More specifically, for α-EuP_3, the crystal structure belongs to the monoclinic space-group type *C*2/*m* and therefore has the point group symmetry of 2/*m*. The *b*-axis is the two-fold rotational axis, and the mirror plane is the *ac*-plane, which is perpendicular to the *b*-axis.

Under magnetic order with ***q*** = (-0.726, 0, 0.222), the magnetic point group symmetry for the helimagnetic phase is expected to be 21'. Note that the prime (') denotes time reversal. By applying a magnetic field (i.e. a ferromagnetic component) along the *a*-axis, the magnetic point group symmetries for the conical and fan phases are expected to be 2' and 2'/*m*', respectively.

(7-b) *q*-vector direction

In the calculation, we adopt ***q*** = (-0.75, 0, 0) while the reported wave vector is ***q*** = (-0.726, 0, 0.222). One might worry that the omission of the *c**-component might cause a qualitatively different result. To assess whether omitting the *c**-component is plausible, we have conducted additional DFT calculations using a ***q***-vector with a finite *c**-component. Since ***q*** = (-0.75, 0, ***0.25***) (i.e., 4 × 1 × 4 supercell) was expected to be too large to calculate within our computational resources, we adopted ***q*** = (-0.75, 0, ***0.5***) (i.e., 4 × 1 × 2 supercell). In Fig. S10 A-E we show the electronic band structures, calculated for the magnetic structure with ***q*** = (-0.75, 0, 0.5) illustrated in panels F and G. The helical plane is set to be perpendicular to the ***q***-vector. While the band diagrams become more complex due to band folding resulting from the larger supercell, the overall symmetry of the band structures for all the helical, conical, and fan phases remains consistent with the results obtained in the original calculation, which omitted the *c**-component of the ***q***-vector. Specifically, the band structures are symmetric in the helical and fan phases, and asymmetric in the conical phase. The noticeable asymmetry in the conical phase is emphasized by the dotted ellipsoids in Fig. S10. These results demonstrate that the *c**-component is not a decisive factor in determining the symmetry that governs the emergence of nonreciprocal transport, thereby justifying the original approximate model structure presented in Fig. S8.

In addition, the plotted -Y - Γ - Y direction is not parallel to the ***q***-vector in this model. The electronic transport direction reflects the band dispersion direction in the momentum space. This calculation also suggests that the slight tilting of the electric current direction from the ***q***-vector does not matter for the selection rule of $\rho^{2\omega}_{\mathrm{asym}}$.

(7-c) Magnetic field direction

In the simplified model, the magnetic field is treated to be parallel to the ***q***-vector, which was not the case in the actual experiment. ***q*** = (-0.726, 0, 0.222) is tilted by ~ 6 degrees towards the *c*-axis shown in Fig. S11.

Let us discuss the effect of magnetic field tilting. In an isotropic helimagnet such as cubic MnSi, the ***q***-vector rotates in an applied magnetic field to align with the magnetic field direction, whereas in anisotropic materials, the ***q***-vector is fixed. The effect of changing the field direction on a helimagnetic structure in an anisotropic material such as monoclinic α-EuP_3 should be mainly the rotation of helical plane. Such kind of helical plane rotations were previously observed in several

helimagnets [S8, S9]. To assess such effect, we have performed band calculations for the helical magnetic structure with tilted helical planes (using $\boldsymbol{q}$ = (-0.75, 0, 0)). The results displayed in Fig. S12 show that the overall features and symmetry are hardly changed by helical plane rotation of ±30 degrees. Note that the angle +17.5 degrees presented in the third panel approximately corresponds to the actual helical plane angle reported in experiment [18].

Fig. S13 further explores the field evolution starting from one of the tilted helical planes. Here too, even in a tilted helical, conical, or fan plane, the original symmetry discussion remains unaffected: the band structure evolves from symmetrical to asymmetrical, and then back to symmetrical.

Section 8 (T, x) phase diagram for $Eu(As_{1-x}P_x)_3$

Fig. S14 displays a (T, x) phase diagram constructed from Brown & Chattopadhyay (1997) [18] and Onuki et al., (2023) [21] along with data for α-EuP_3 (This work and Mayo et al., (2022) [14]). The transition temperatures for α-EuP_3 (i.e., x = 1) follows the trend seen in the previous reports of $Eu(As_{1-x}P_x)_3$ (x = 0 – 0.98).

Section 9 $\rho^{2\omega}_{asym}$ measured by subsequently sweeping the magnetic field between -4T and +4T at 4.2 K

Fig. S15 shows the raw (A) and field-asymmetrized (B) data of $\rho^{2\omega}$ at T = 4.2 K, measured by subsequently sweeping the magnetic field between +4 T and -4 T without any poling procedure. For the blue and orange data, the sign of chirality seemed to be switched during the magnetic field sweep. The sign and magnitude were not reproducible across the repeated field sweeps traversing chiral and achiral phases, as expected from uncontrolled chiral domain populations. Nevertheless, a finite $\rho^{2\omega}_{asym}$ is distinctly observed only within the field range of the chiral magnetic phase.

Section 10 Examination of heating effects in $\rho^{2\omega}_{asym}$

One might be concerned that the oscillation of resistivity with increasing/decreasing temperature causes a $\rho^{2\omega}_{asym}$ signal. In that case, $\rho^{2\omega}_{asym}$ would be proportional to the temperature derivative of resistivity. We measured the magnetic field dependence of resistivity $\rho^{1\omega}$ at various temperatures (T) and estimated $d\rho^{1\omega}/dT$ as $[\rho^{1\omega}(T+\Delta T) - \rho^{1\omega}(T)]/\Delta T = \Delta\rho^{1\omega}/\Delta T$. The result is shown in Fig. S16. The magnetic field dependence of $\Delta\rho^{1\omega}/\Delta T$ does not resemble that of $\rho^{2\omega}_{asym}$ for any temperature range. It can be also noted that $\Delta\rho^{1\omega}/\Delta T$ shows features even in the PM phase, where the $\rho^{2\omega}_{asym}$ is absent. Therefore, the heating mechanism seems to be unlikely.

In addition, the heating effect cannot explain the random emergence of positive/negative $\rho^{2\omega}_{asym}$. Because linear resistivity is independent of chirality, the second harmonic signal originating from the combination of heating and temperature-dependent resistivity should have a unique sign. In addition, Hall and Nernst signals are also independent of chirality. While it has been discussed how spin-chirality $S_i \cdot (Sj \times S_k)$ can induce a Hall resistivity (for example, [S10]), the notion of chirality

we present in this work is related to the mirror symmetry breaking and is clearly distinct from the spin-chirality. In principle, chirality cannot be probed by any linear transport. Therefore, any second harmonic signal related to the Nernst effect should also have a unique sign and thus cannot explain the observation. In addition, these extrinsic origins cannot explain the disappearance of the second harmonic signal above the helical transition temperature.

Section 11 Frequency and AC current dependences of $\rho^{2\omega}_{asym}$

Frequency and ac current dependences of $\rho^{2\omega}_{asym}$ are shown in Fig. S17. $\rho^{2\omega}_{asym}$ proves to be independent in the frequency range of 5.15 – 79.15 Hz (Fig. S17A), and shows a linear j_0 dependence (Figs. S17B and C), as expected from $\rho^{2\omega} = \rho^2 j_0/2$ (Eq. (2)).

Section 12 Alignment of crystal axes for the FIB sample

Figs. S18 and S19 describe how the crystal was aligned for the FIB device. It was confirmed through single crystal x-ray diffraction in larger crystals that α-EuP_3 single crystals consistently have a distinct facet with a bright metallic luster showing the *ab*-plane, along with a characteristic pattern where there are lines strictly parallel to the *b*-axis (Fig. S18). These features enable us to identify the crystal orientations using an optical microscope or SEM observations.

For the FIB device, we picked a small thin flake showing the *ab*-plane facet and stripe patterns, placed it on an oxidized single-crystal silicon wafer, deposited Pt/Au, and then conducted FIB fabrication (the photographs of the FIB process in Fig. S19 show the exact same crystal used for the device in Fig. 2A). We also attempted to cut out a lamella from the bulk using FIB, but this approach resulted in poor electrode contacts. Given that the material is a low-carrier semimetal, even slight carrier doping or damage at the sample/electrode interface caused by exposure to the Ga-FIB could be detrimental to achieving ohmic contact. The process described in Fig. S19 ensures that ohmic contact is established before any exposure to the FIB, thus avoiding this issue.

Section 13 Terminal dependence of first and second harmonic resistance

In Fig. S20, we show the first and second harmonics of the two-terminal resistance (terminals 1-6), as well as the first and second harmonics of the four-terminal resistances (terminals 2-3, 3-4, and 4-5). The two-terminal resistance does not show significant field asymmetry even though the simultaneously measured four-terminal resistances display the characteristic asymmetry discussed in the main manuscript, proving that extrinsic effects from electrode contacts are negligible.

Interestingly, V_{23} and V_{45} clearly reproduce the characteristic field dependence, but with opposite signs, while V_{34} shows a more subtle signal. This voltage terminal dependence can be understood as the result of probing different chiral domain populations across the sample: one chirality domain is dominant between terminals 2 and 3, while the opposite chirality domain is dominant between

terminals 4 and 5. Between terminals 3 and 4, the opposing domains are presumably cancelling each other out, resulting in a smaller signal.

Section 14 Comparison between the bulk and FIB samples

In Fig. S21, we compare the data of bulk and FIB samples. To distinguish these samples from the bulk sample presented in Fig. 4, whose configuration was $I \perp a$, we label these bulk samples (I // a) as Bulk sample No. 2 and No.3. For the FIB samples, the sample presented throughout the main text and shown in Fig. 2A is referred to as FIB sample No. 1 and the spot-welded sample shown in Fig. S3A is labeled FIB sample No. 2.

Sample dependences are discerned in the magnitude of the resistivities, regardless of whether they are bulk or FIB-fabricated samples. However, crucial features are consistent throughout all samples, such as the metallic temperature dependence, two magnetic phase transitions at low temperatures, and a kink in the field dependence followed by a negative MR.

Fig. S1.

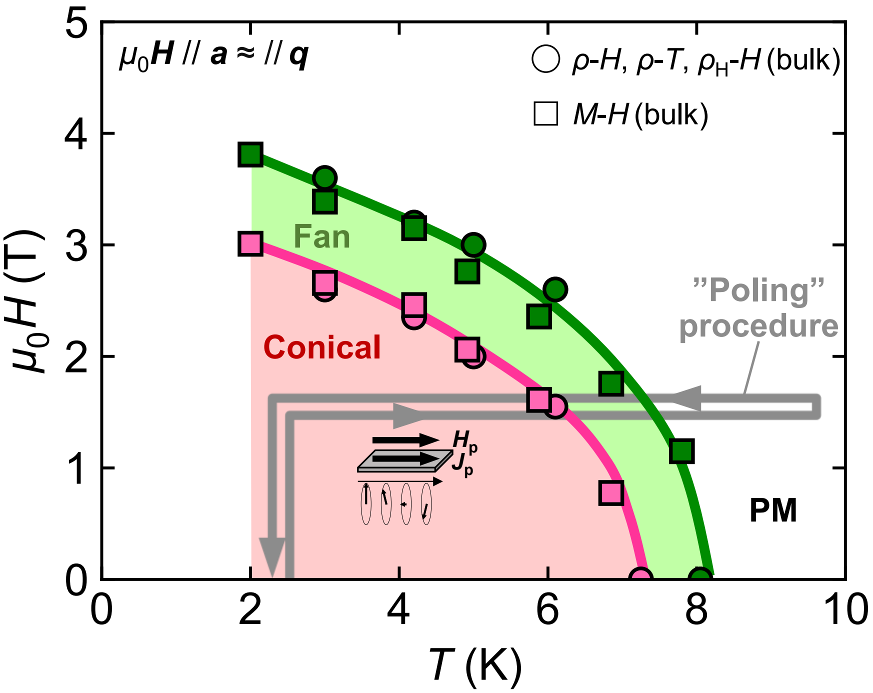

Relation between the chirality-control procedure profile and magnetic phase diagram. Diagram of the chirality-control (poling) procedure combining an external magnetic field H_p and a dc electric current density J_p. The poling procedure is denoted as a grey path traversing the magnetic phases. The temperature sweep is done by sweeping the dc electric current and utilizing the Joule heating of the sample. The inset schematic illustrates the experimental configuration for the parallel poling ($H_p > 0$, $J_p > 0$) case.

Fig. S2.

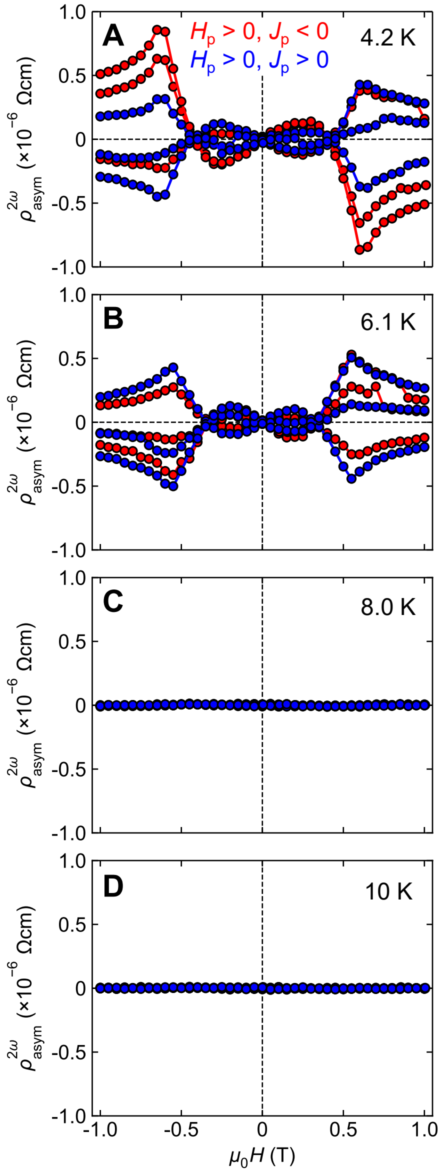

Field-asymmetric component of the second-harmonic resistivity $\rho^{2\omega}{}_{asym}$ at various temperatures. Magnetic field dependence of $\rho^{2\omega}{}_{asym}$ measured after the poling procedures with parallel ($H_p > 0$, $J_p > 0$) and anti-parallel ($H_p > 0$, $J_p < 0$) magnetic field and electric current at (**A**) T = 4.2 K, (**B**) 6.1 K, (**C**) 8.0 K, and (**D**) 10 K. $\rho^{2\omega}{}_{asym}$ was measured three times for both $H_p > 0$, $J_p >$ 0 and $H_p > 0$, $J_p < 0$ poling procedures at each temperature. The temperatures correspond to the helical/conical (4.2 K and 6.1 K), sinusoidal/fan (8.0 K), and paramagnetic (10 K) phases. While we observe finite $\rho^{2\omega}{}_{asym}$ at 4.2 K and 6.1 K, the sign and magnitude are not reproduced.

Fig. S3.

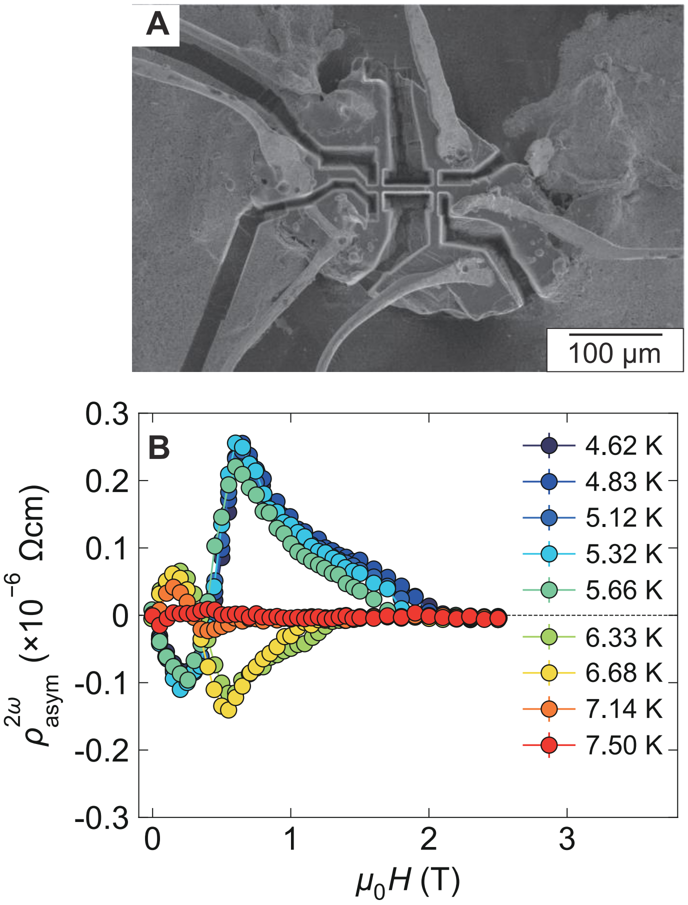

Magnetic field dependence of $\rho^{2\omega}{}_{asym}$ measured in a different sample. (**A**) SEM image of a microfabricated sample. (**B**) Isotherms of the magnetic field dependence of $\rho^{2\omega}{}_{asym}$ measured in the microfabricated sample shown in (**A**). This sample is denoted as FIB sample No. 2.

Fig. S4.

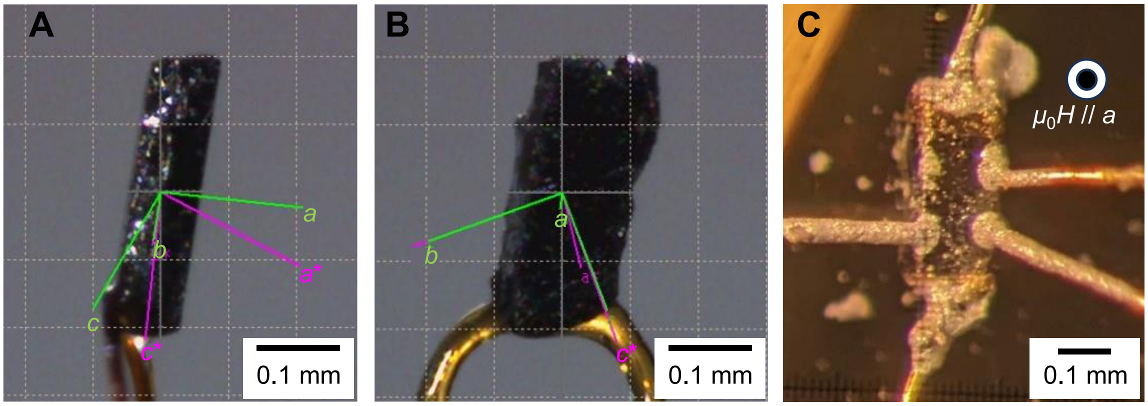

Resistivity measurement configuration on a bulk sample. Photographs of the sample used for bulk resistivity measurements. (**A**) and (**B**) display the crystal orientation obtained from single crystal x-ray analysis. (**C**) shows the resistivity measurement configuration.

Fig. S5.

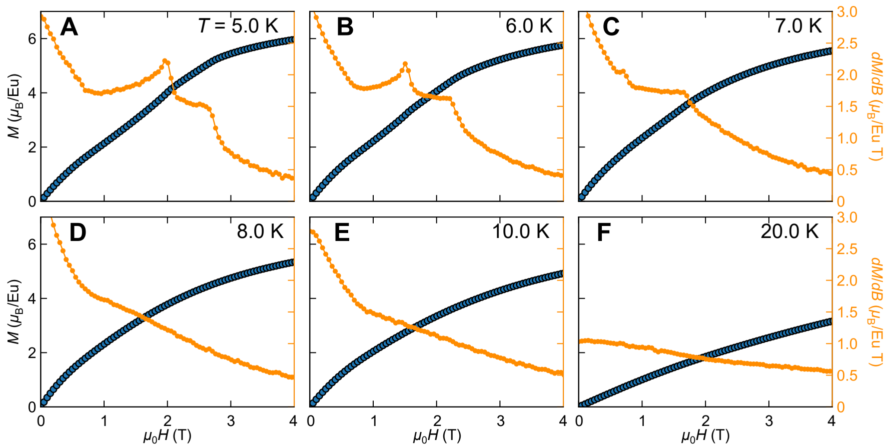

Magnetic field dependence of magnetization. (**A**)-(**F**) show the magnetic field dependence of the magnetization and its field derivative at various temperatures up to 20 K measured in a bulk sample.

Fig. S6.

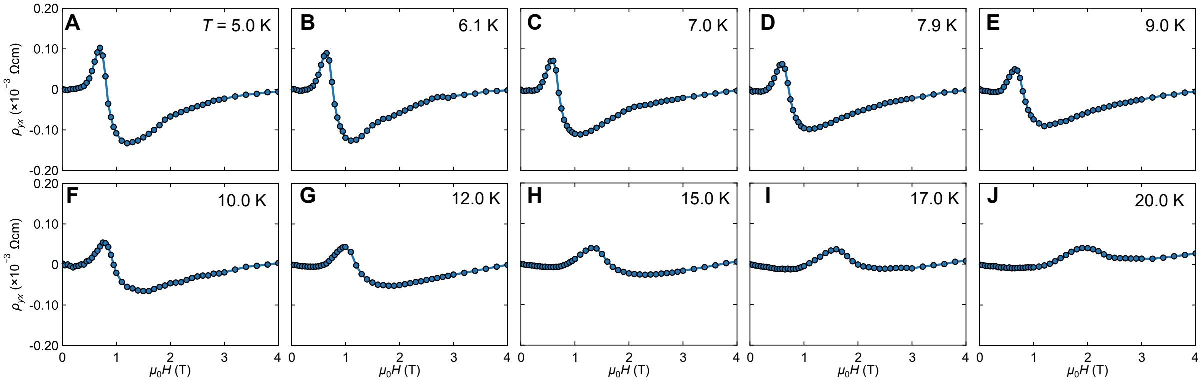

Magnetic field dependence of the Hall resistivity. (**A**)-(**J**) Magnetic field dependence of the Hall resistivity at various temperatures up to 20 K measured in a bulk sample.

Fig. S7.

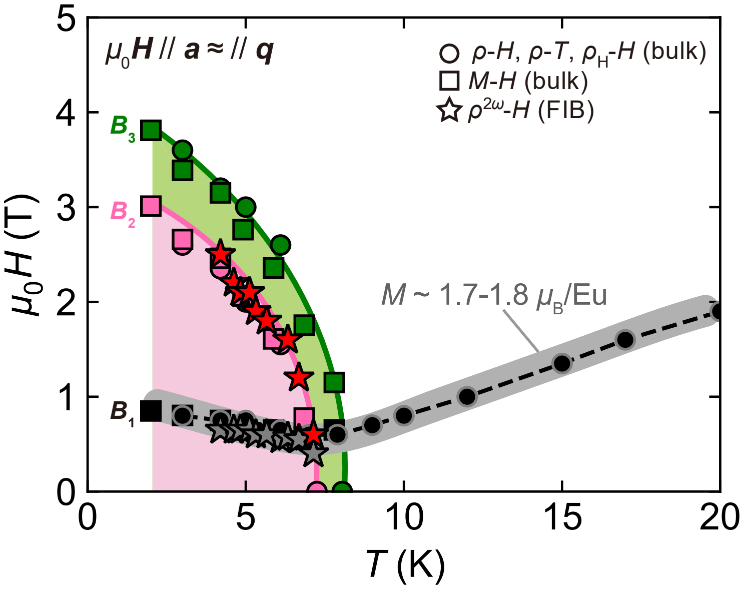

Magnetic phase diagram and resistivity anomalies. Field-temperature phase diagram of α-EuP_3 for a magnetic field $\mu_0\boldsymbol{H}$ // $\boldsymbol{a}$, along with the Hall resistivity anomalies shown up to T = 20 K. B_1, B_2, and B_3 denotes the magnetic fields where the anomalies and phase transitions occur as shown in Figure 3.

Fig. S8.

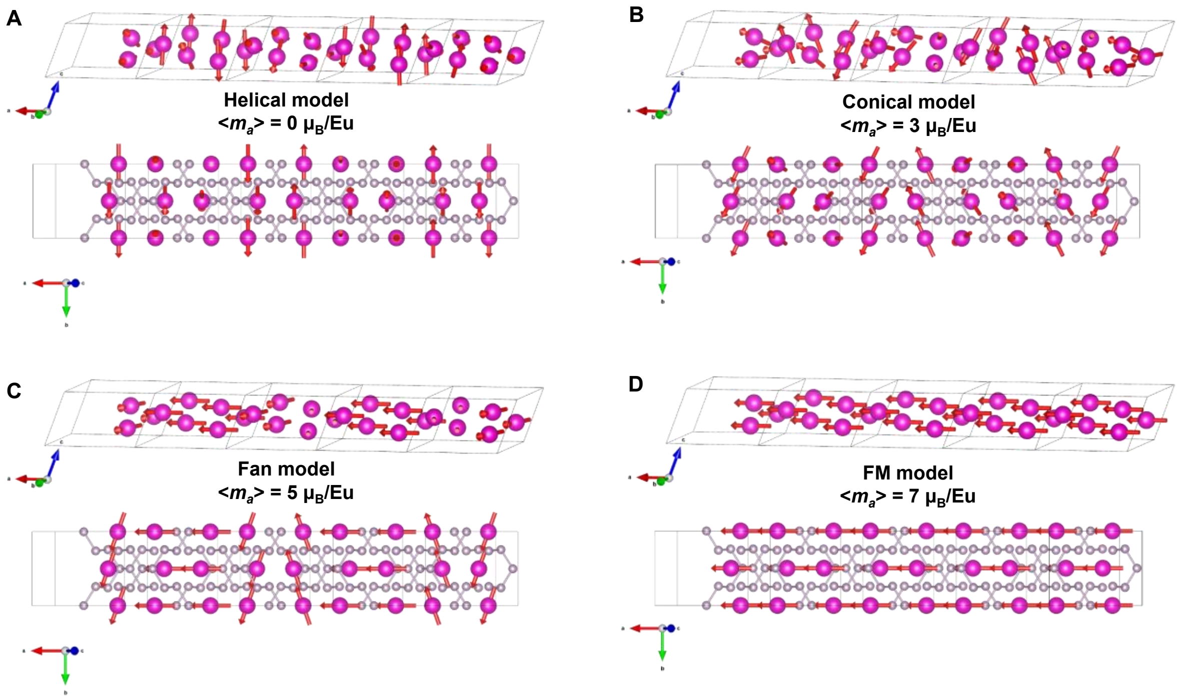

Simplified magnetic models assuming a propagation vector of q = (-0.75, 0, 0). Arrangement of the Eu moments in the (**A**) helimagnetic model, (**B**) conical model, (**C**) fan model, and (**D**) ferromagnetic model. Panel **B** shows the case for 3 μ_B/Eu as one example for the conical structure. Only Eu ions are depicted in the top panels for simplicity.

Fig. S9.

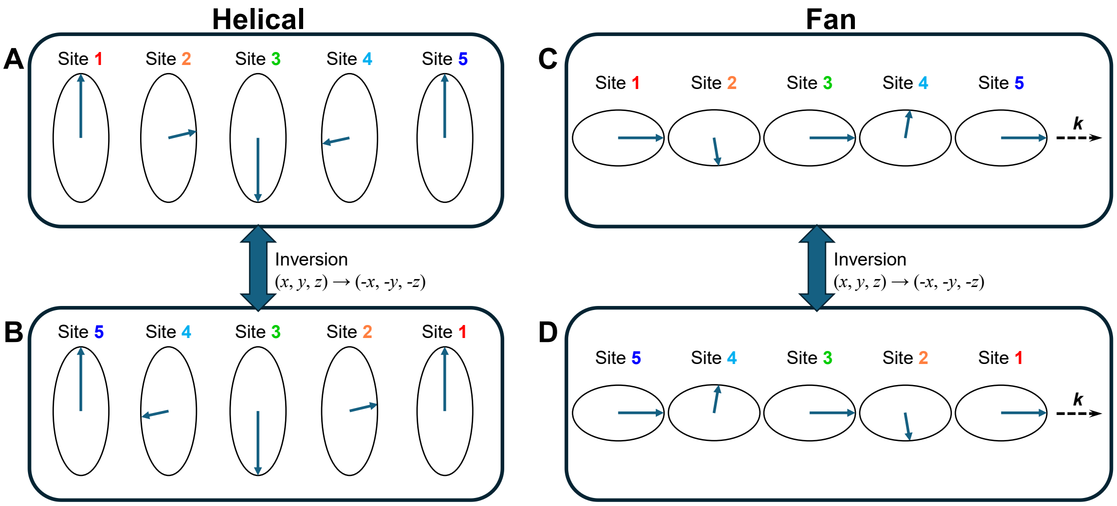

Effect of inversion on helical and fan structures. For the helimagnetic structure, spatial inversion converts the system to the opposite enantiomer (from **A** to **B**). For the fan structure (**C**), spatial inversion merely results in a translation of the original structure along the depicted *k*-direction (**D**) (In this example the translation is half a period).

Fig. S10.

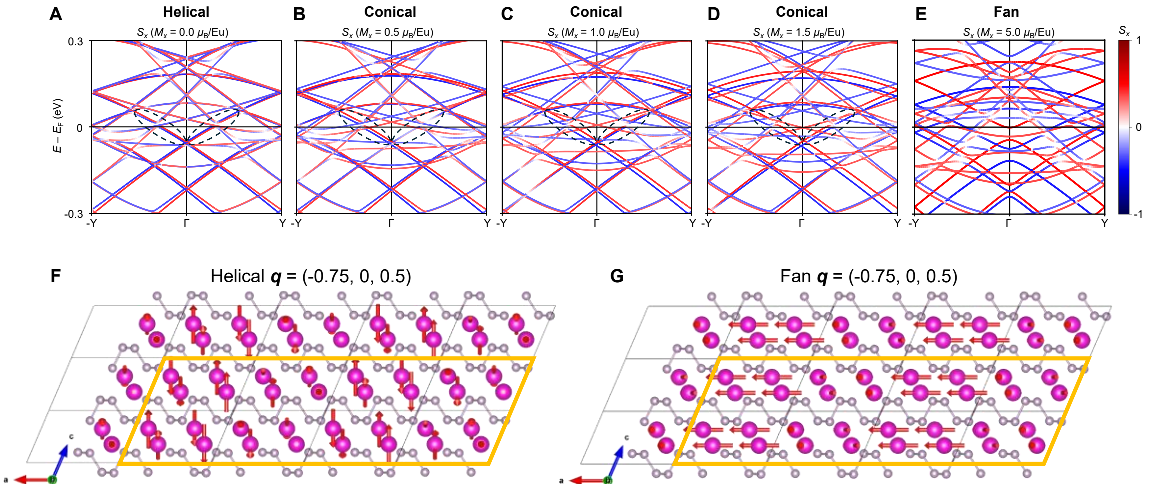

DFT calculations for a *q*-vector with a finite *c-component.** Band structure calculations of (**A**) helical, (**B**, **C**, **D**) conical, and (**E**) fan states, with a magnetic propagation vector of $\boldsymbol{q}$ = (-0.75, 0, 0.5). The noticeable asymmetry in the conical phase is emphasized by the dotted ellipsoids. The magnetic models used in the calculations are depicted in panels **F** and **G** for the helical(conical) and fan states, respectively. The yellow outline in **F** and **G** describes the 4 × 1 × 2 supercell.

Fig. S11.

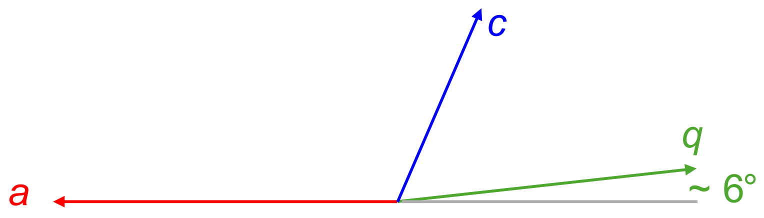

Crystal axes and *q*-vector in α-EuP_3. Schematic of the relative orientations of the crystal axes and the reported ***q***-vector ***q*** = (-0.726, 0, 0.222).

Fig. S12.

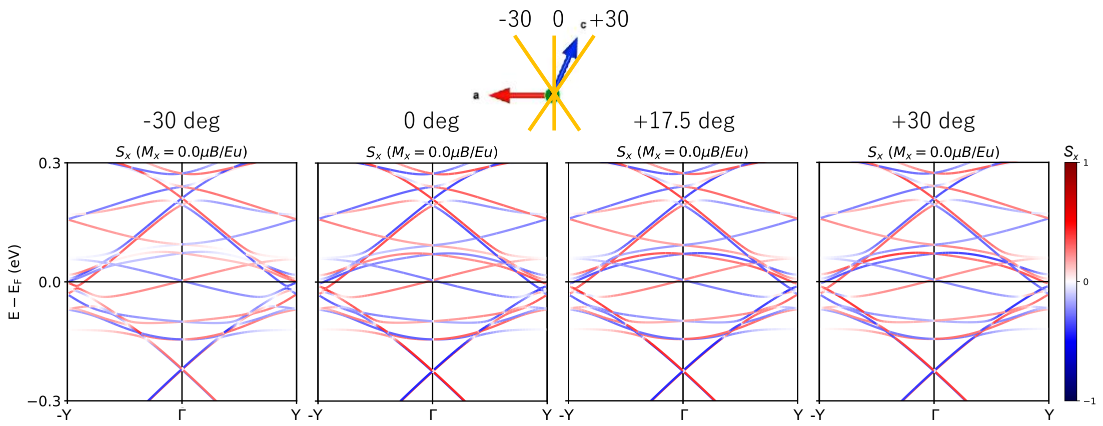

DFT calculations for tilted helical planes. Helical plane tilting dependence computed for approximated ***q***-vector ***q*** = (-0.75, 0, 0). Note that +17.5 degrees approximately corresponds to the experimentally reported helical plane orientation.

Fig. S13.

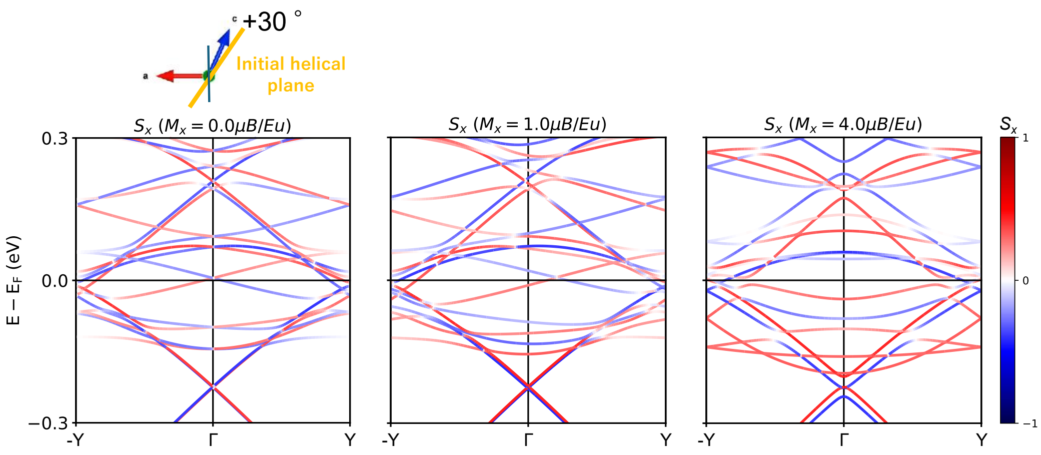

Field evolution calculated for tilted structures. Band structure computed for tilted helical, conical, and fan structures in the approximated ***q***-vector ***q*** = (-0.75, 0, 0).

Fig. S14.

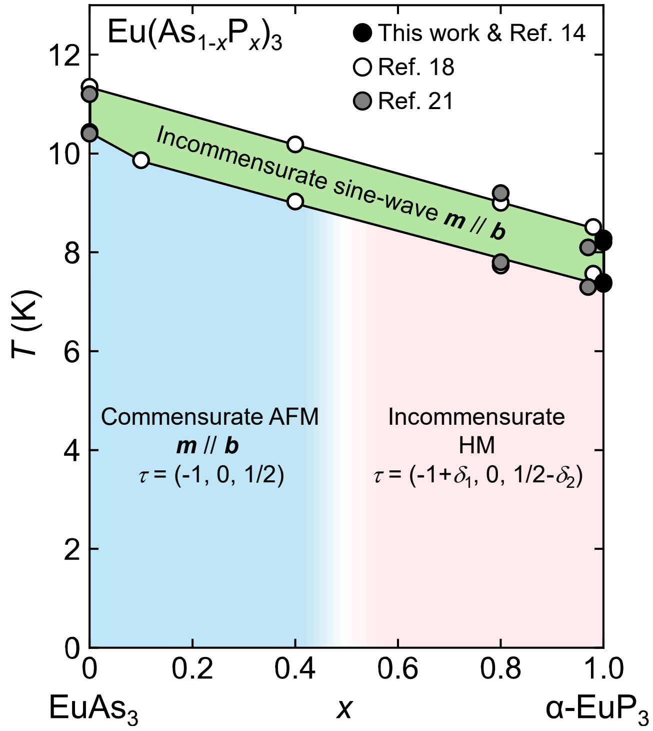

Magnetic phases with varying pnictide compositions. Magnetic phase diagram of $Eu(As_{1-x}P_x)_3$ as a function of temperature T and phosphorus concentration x.

Fig. S15.

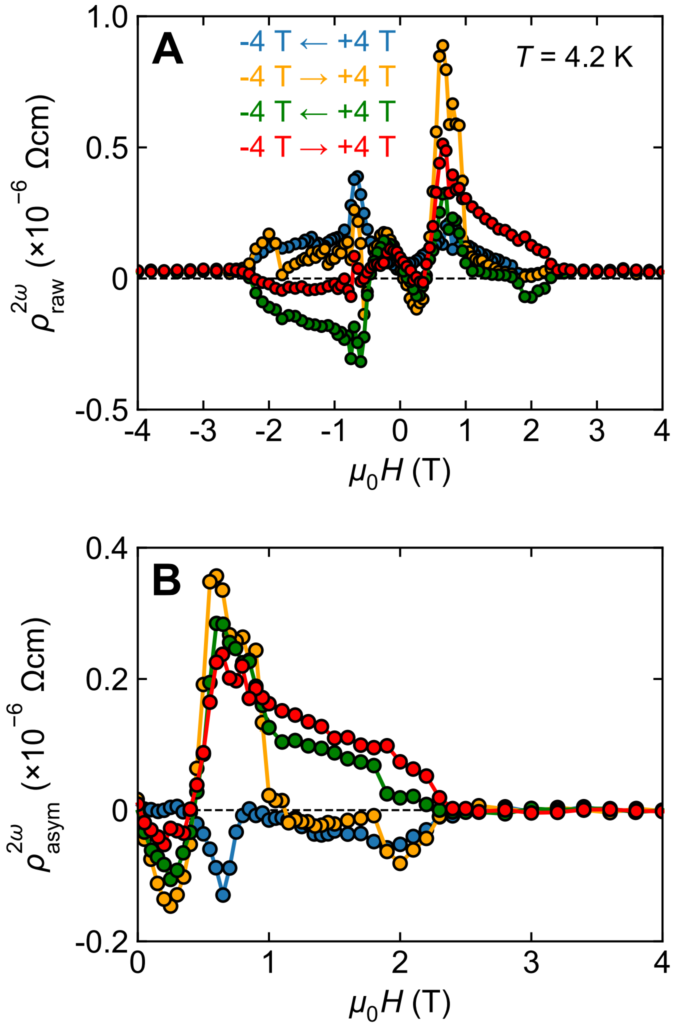

$\rho^{2\omega}$ measurement for repeated field sweeps up to high fields. (**A**) Raw ($\rho^{2\omega}{}_{\mathrm{raw}}$) and (**B**) field-asymmetrized ($\rho^{2\omega}{}_{\mathrm{asym}}$) data of $\rho^{2\omega}$ at T = 4.2 K, measured by subsequently sweeping the magnetic field between +4 T and -4 T, without any poling procedure.

Fig. S16.

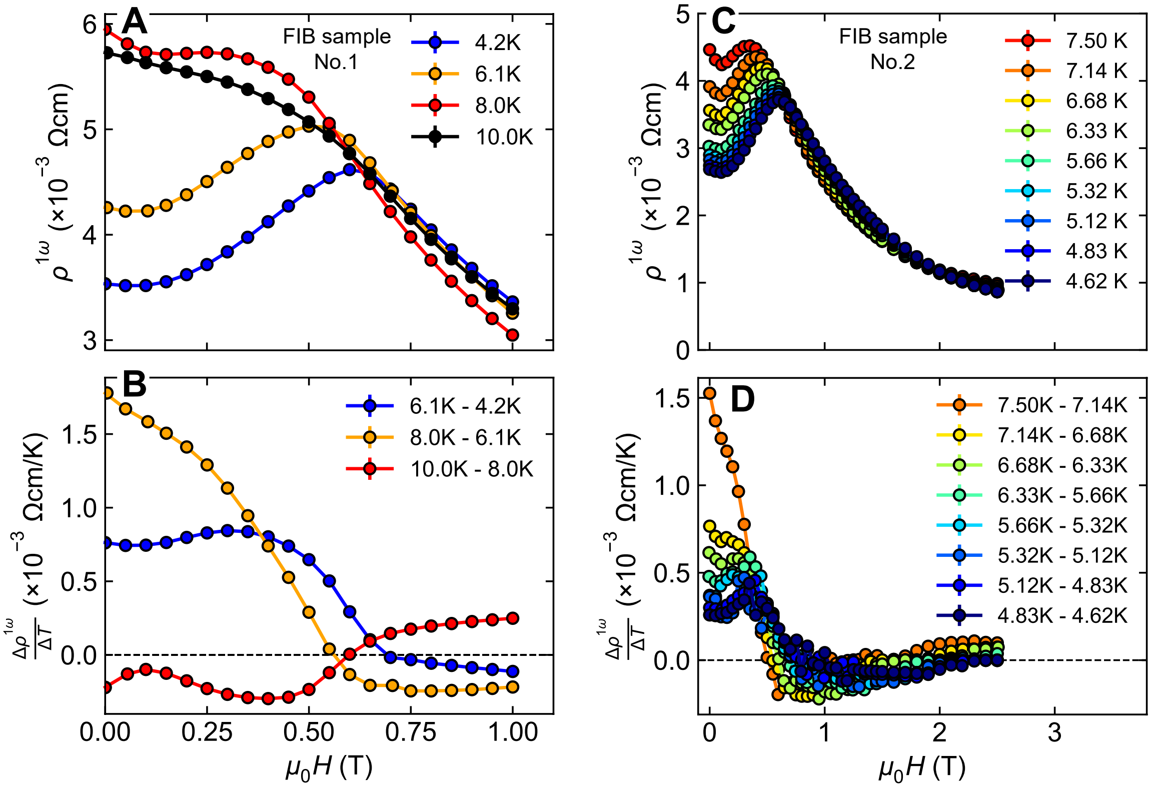

Assessment of heating effects in FIB samples. Field scans of (**A, C**) $\rho^{1\omega}$ and (**B**, **D**) $\Delta\rho^{1\omega}/\Delta T$, measured in the FIB devices FIB sample No.1 and No.2, respectively. FIB sample No. 1 corresponds to the sample presented in the main paper and shown in Fig. 2A, while FIB sample No.2 is shown in Fig. S3A.

Fig. S17.

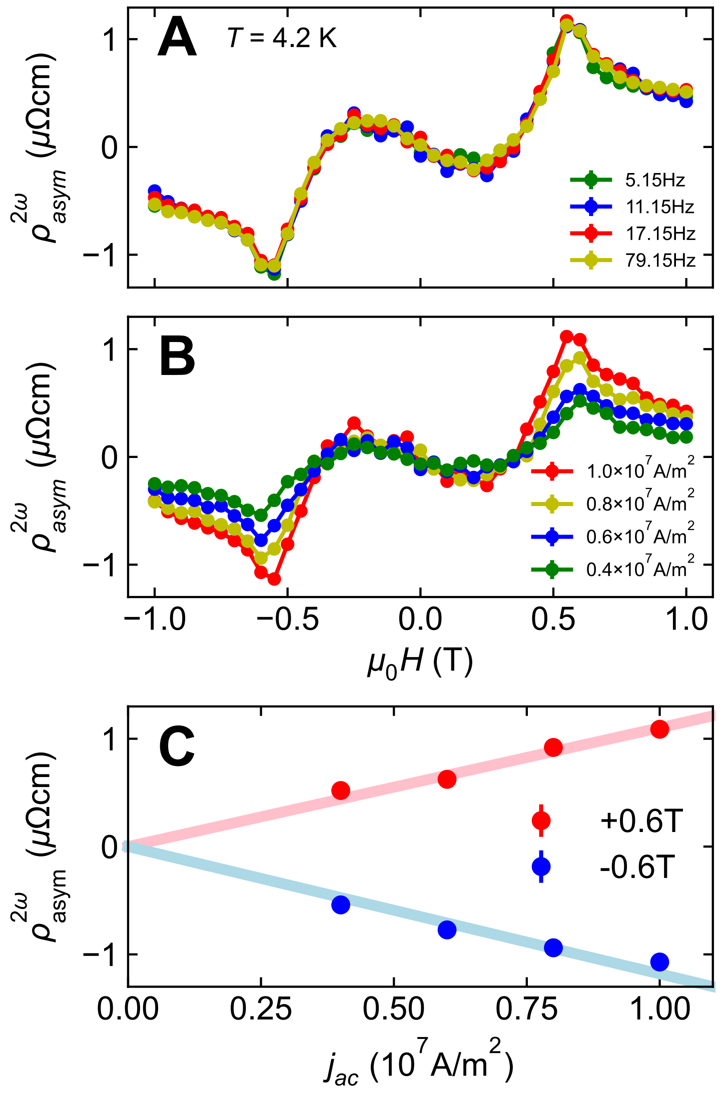

Frequency and ac current dependences of $\rho^{2\omega}{}_{\mathrm{asym}}$. (**A**) Frequency and (**B**, **C**) electric current density dependence of $\rho^{2\omega}{}_{\mathrm{asym}}$ measured at 4.2 K.

Fig. S18.

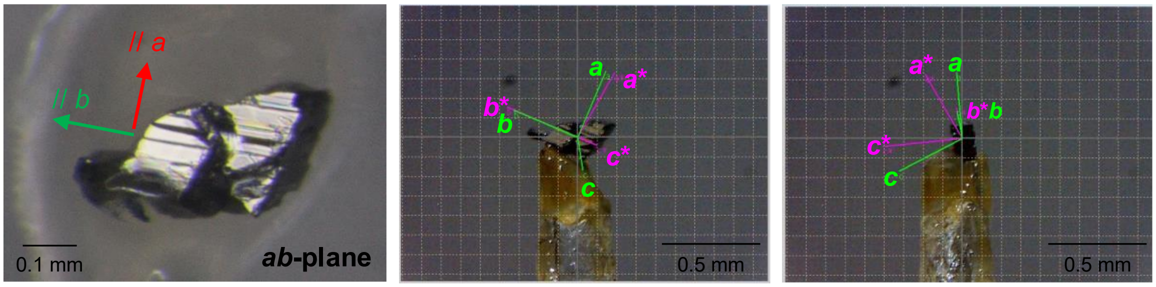

Crystal orientation of bulk crystalline α-EuP_3. Photographs of the *ab*-plane of a bulk α-EuP_3 crystal and its orientation determined by single crystal x-ray diffraction. The green (pink) axes depicted in the latter two panels are the real (reciprocal) axes refined from the analysis.

Fig. S19.

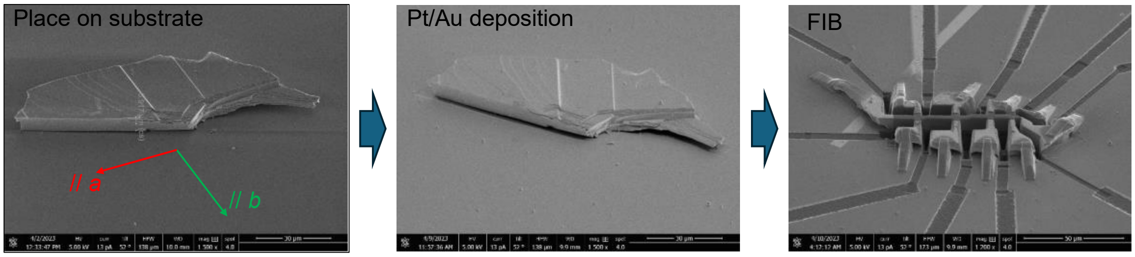

Fabrication process of the FIB device presented in Figure 2A. To assure ohmic electrode contacts, the FIB device presented in the main text was fabricated by first depositing Pt/Au on the entire crystal before any exposure to the FIB, and then shaped into a Hall bar with FIB.

Fig. S20.

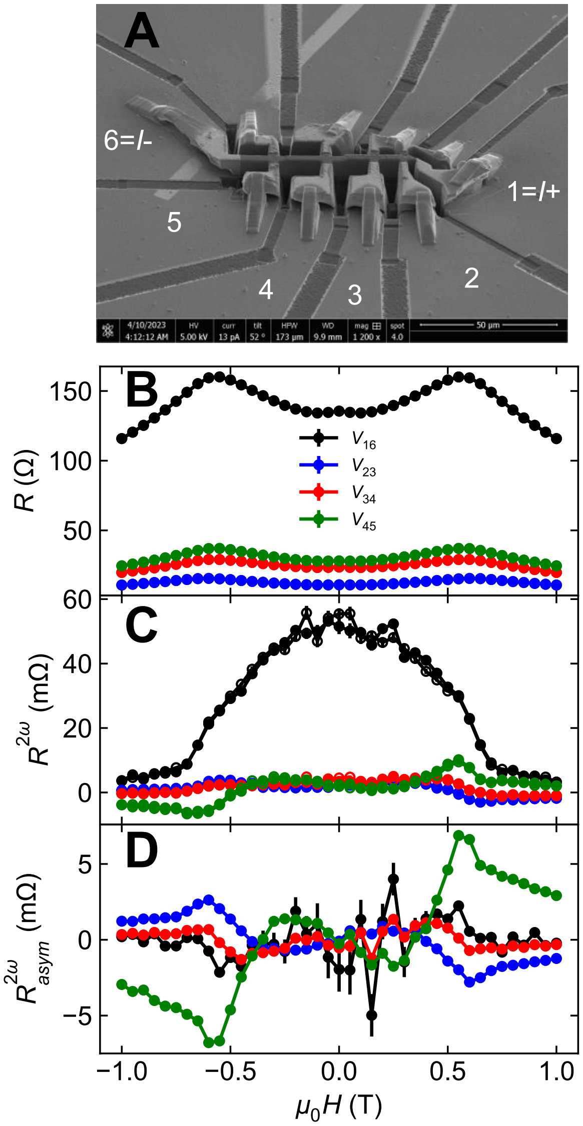

Terminal dependence of first and second harmonic resistance. (**A**) SEM image of the FIB device. The terminals used for the voltage measurement are labeled with numbers from 1 to 6. Terminals 1 and 6 are for the two-terminal measurement and terminals 2,3,4, and 5 are for the four-terminal measurements. (**B**) First-harmonic resistances for the two-terminal and four-terminal contacts. (**C**) Second-harmonic resistances for the two-terminal and four-terminal contacts. (**D**) Field-asymmetric components obtained from the two-terminal and four-terminal measurements.

Fig. S21.

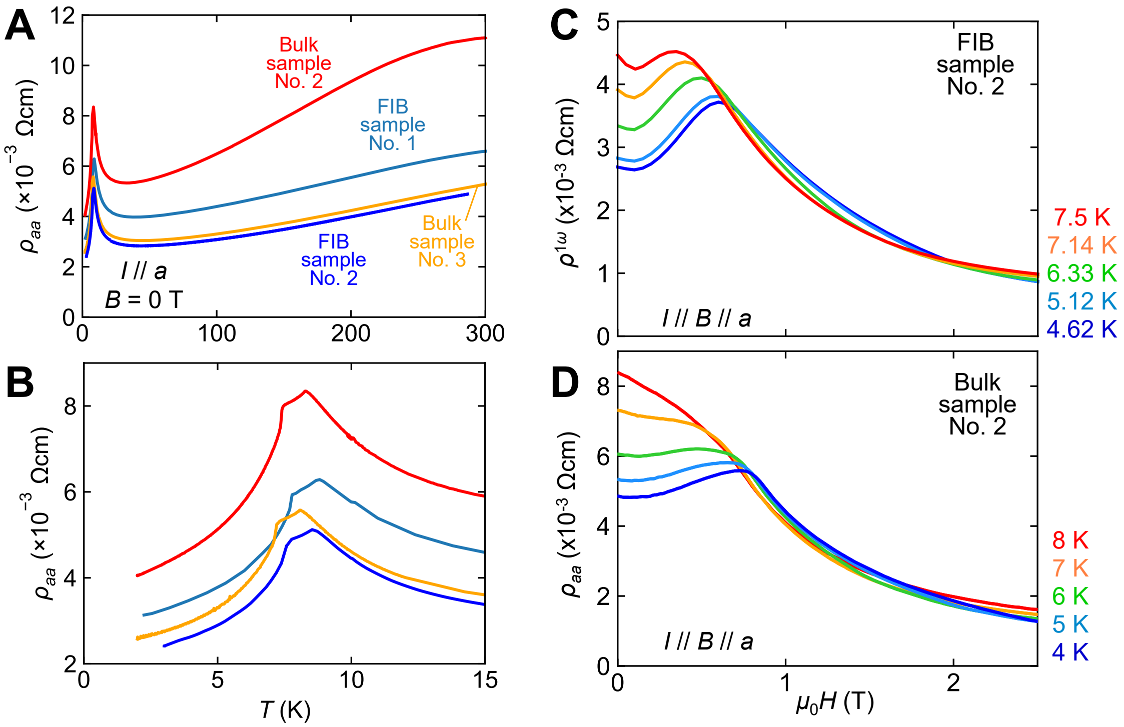

Comparison between bulk and FIB samples. (**A**) Temperature dependence of resistivity measured with *I* // *a* in bulk crystals and FIB devices. (**B**) Low temperature behavior in panel A. (**C** and **D**) Magnetic field dependence of the longitudinal resistivity for (**C**) FIB sample No.1 and (**D**) Bulk sample No. 2, respectively. The bulk samples used for these figures are different from the bulk sample presented in Fig. 4, whose configuration was *I* ⊥ *a*. We label the bulk samples presented here (*I* // *a*) as Bulk samples No. 2 and No.3. For FIB samples, FIB sample No. 1 is the sample shown in Fig. 2A and FIB sample No. 2 is the sample shown in Fig. S3A.

Table S1.
List of the magnetochiral anisotropy coefficient γ for various published materials. TSM and RT stand for topological semimetal and room temperature, respectively. Note that there is a detailed discussion regarding the $\mathscr{P}$-breaking in $ZrTe_5$[S5].

**Material**	**$|\gamma|$ ($m^2T^{-1}A^{-1}$)**	**Crystal structure**	**Note**	**Reference**
$ZrTe_5$	4×10^{-7}	Polar	TSM, T = 3 K	[S5]
WTe_2	3.4×10^{-7}	Polar	TSM, T = 5 K	[S6]
BiTeBr	3×10^{-12}	Polar	T = 2 K	[13]
Te	10^{-8}	Chiral	RT	[S7]
$CrNb_3S_6$	10^{-12}	Chiral	Magnetic, T = 90 K	[5]
MnSi	2×10^{-13}	Chiral	Magnetic, T = 35 K	[4]
MnP	4×10^{-13}	Centrosymmetric	Magnetic, T = 51 K	[6]
$MnAu_2$	2×10^{-14}	Centrosymmetric	Magnetic, RT	[7]
**α-EuP_3**	**6×10^{-11}**	**Centrosymmetric**	**Magnetic, T = 4.2 K**	**This work**

SI References

S1. A. Yanase, A. Hasegawa, Electronic structure of MnP. *J. Phys. C: Solid State Phys.* **13**, 1989 (1980).

S2. J. Xiao, A. Zangwill, M. D. Stiles, Macrospin models of spin transfer dynamics. *Phys. Rev. B* **72**, 014446 (2005).

S3. O. Wessely, B. Skubic, L. Nordström, Spin-transfer torque in helical spin-density waves. *Phys. Rev. B* **79**, 104433 (2009).

S4. V. V. Ustinov, I. A. Yasyulevich, Chirality-dependent spin-transfer torque and current-induced spin rotation in helimagnets. *Phys. Rev. B* **106**, 064417 (2022).

S5. Y. Wang, H. F. Legg, T. Bömerich, J. Park, S. Biesenkamp, A. A. Taskin, M. Braden, A. Rosch, Y. Ando, Gigantic Magnetochiral Anisotropy in the Topological Semimetal $ZrTe_5$. *Phys. Rev. Lett.* **128**, 176602 (2022).

S6. T. Yokouchi, Y. Ikeda, T. Morimoto, Y. Shiomi, Giant Magnetochiral Anisotropy in Weyl Semimetal WTe_2 Induced by Diverging Berry Curvature. *Phys. Rev. Lett.* **130**, 136301 (2023).

S7. G. L. J. A. Rikken, N. Avarvari, Strong electrical magnetochiral anisotropy in tellurium. *Phys. Rev. B* **99**, 245153 (2019).

S8. S. Ishiwata, Y. Taguchi, H. Murakawa, Y. Onose, Y. Tokura, Low-Magnetic-Field Control of Electric Polarization Vector in a Helimagnet. *Science* **319**, 1643 (2008).

S9. H. Murakawa, Y. Onose, F. Kagawa, S. Ishiwata, Y. Kaneko, Y. Tokura, Rotation of an Electric Polarization Vector by Rotating Magnetic Field in Cycloidal Magnet $Eu_{0.55}Y_{0.45}MnO_3$, *Phys. Rev. Lett.* **101**, 197207 (2008).

S10. Y. Taguchi, Y. Oohara, H. Yoshizawa, N. Nagaosa, Y. Tokura, Spin chirality, Berry phase, and anomalous Hall effect in a frustrated ferromagnet, *Science* **291**, 2573 (2001).
